# The expression characteristics of transmembrane protein genes in pancreatic ductal adenocarcinoma through comprehensive analysis of bulk and single-cell RNA sequence

**DOI:** 10.3389/fonc.2023.1047377

**Published:** 2023-05-17

**Authors:** Chen Ye, Siqian Ren, Abuduhaibaier Sadula, Xin Guo, Meng Yuan, Meng Meng, Gang Li, Xiaowei Zhang, Chunhui Yuan

**Affiliations:** ^1^ Department of General Surgery, Peking University Third Hospital, Beijing, China; ^2^ Department of Hepatobiliary surgery, Beijing Chaoyang Hospital, Capital Medical University, Beijing, China; ^3^ Department of Hematology, Beijing Shijitan Hospital, Capital Medical University, Beijing, China

**Keywords:** transmembrane protein gene, pancreatic ductal adenocarcinoma, tumor microenvironment, single-cell RNA sequence, prognostic marker

## Abstract

**Background:**

Transmembrane (TMEM) protein genes are a class of proteins that spans membranes and function to many physiological processes. However, there is very little known about TMEM gene expression, especially in cancer tissue. Using single-cell and bulk RNA sequence may facilitate the understanding of this poorly characterized protein genes in PDAC.

**Methods:**

We selected the TMEM family genes through the Human Protein Atlas and characterized their expression by single-cell and bulk transcriptomic datasets. Identification of the key TMEM genes was performed through three machine learning algorithms: LASSO, SVM-RFE and RF-SRC. Then, we established TMEM gene riskscore and estimate its implication in predicting survival and response to systematic therapy. Additionally, we explored the difference and impact of TMEM gene expression in PDAC through immunohistochemistry and cell line research.

**Results:**

5 key TMEM genes (ANO1, TMEM59, TMEM204, TMEM205, TMEM92) were selected based on the single-cell analysis and machine learning survival outcomes. Patients stratified into the high and low-risk groups based on TMEM riskscore, were observed with distinct overall survival in internal and external datasets. Moreover, through bulk RNA-sequence and immunohistochemical staining we verified the protein expression of TMEM genes in PDAC and revealed TMEM92 as an essential regulator of pancreatic cancer cell proliferation, migration, and invasion.

**Conclusion:**

Our study on TMEM gene expression and behavior in PDAC has revealed unique characteristics, offering potential for precise therapeutic approaches. Insights into molecular mechanisms expand understanding of PDAC complexity and TMEM gene roles. Such knowledge may inform targeted therapy development, benefiting patients.

## 1 Introduction

Pancreatic ductal adenocarcinoma (PDAC) is an extremely aggressive malignancy, responsible for more than 40,000 deaths annually worldwide (1, 2). Although surgical resection of the primary tumor remains the best chance for a cure, this option is only available to approximately 30% of patients due to locally advanced or metastatic disease (3). Multidisciplinary approaches that combine neoadjuvant treatment and postoperative chemotherapy have facilitated radical resection and prolonged tumor-free survival in PDAC patients (4). Despite this, overall survival rates in resectable PDAC have not significantly improved. Numerous studies have analyzed the molecular heterogeneity of PDAC and identified various subtypes, which can contribute to totally divergent biological behavior and prognostic impact (5, 6). Further investigation into the gene expression characteristics of PDAC may provide insight into the underlying genetic alterations and extend the potential therapeutic targets for this deadly disease (4).

Transmembrane (TMEM) proteins are a class of proteins that span the entire width of the lipid bilayer membrane, and participate in a wide range of physiological processes (7). While some TMEM proteins have been extensively studied and found to be involved in transmembrane transport, signal transduction, apoptosis, and autophagy, others remain poorly characterized in terms of their structure, biological function, and mechanism of action (8). Some TMEM proteins have even been reclassified into more specific categories, such as ANO proteins, after further characterization (9).

Notably, abnormal expression of TMEM genes in cancer has been linked to distant metastases and tumor recurrence, suggesting a potential role in cancer pathogenesis (10, 11). Despite this association, little is known about the expression and biological function of TMEM genes in the tumor microenvironment. Therefore, we speculate that identifying the expression pattern of TMEM genes could potentially shed light on their roles in the complex landscape of the tumor microenvironment (12). A deeper understanding of these proteins and their functions in cancer could pave the way for novel therapeutic strategies in the fight against this devastating disease.

This article provides a comprehensive investigation of TMEM genes in normal pancreas and PDAC tissues. Using clinical information and expression data from The Cancer Genome Atlas Project, we performed a prognostic analysis of TMEM gene expression in bulk tissue samples. Next, we utilized single-cell RNA sequencing to explore the expression patterns of TMEM genes in different cells of the PDAC tumor microenvironment, including tumor, stromal, and immune cells. Additionally, we employed a range of methods, including bulk RNA sequencing, immunohistochemistry staining, and cell-based assays, to validate our findings. Our results indicate that a comprehensive analysis of TMEM genes could help elucidate their function in different cells within the tumor stroma and their prognostic impact in PDAC.

## 2 Materials and methods

### 2.1 Data collection

**Figure 1:**
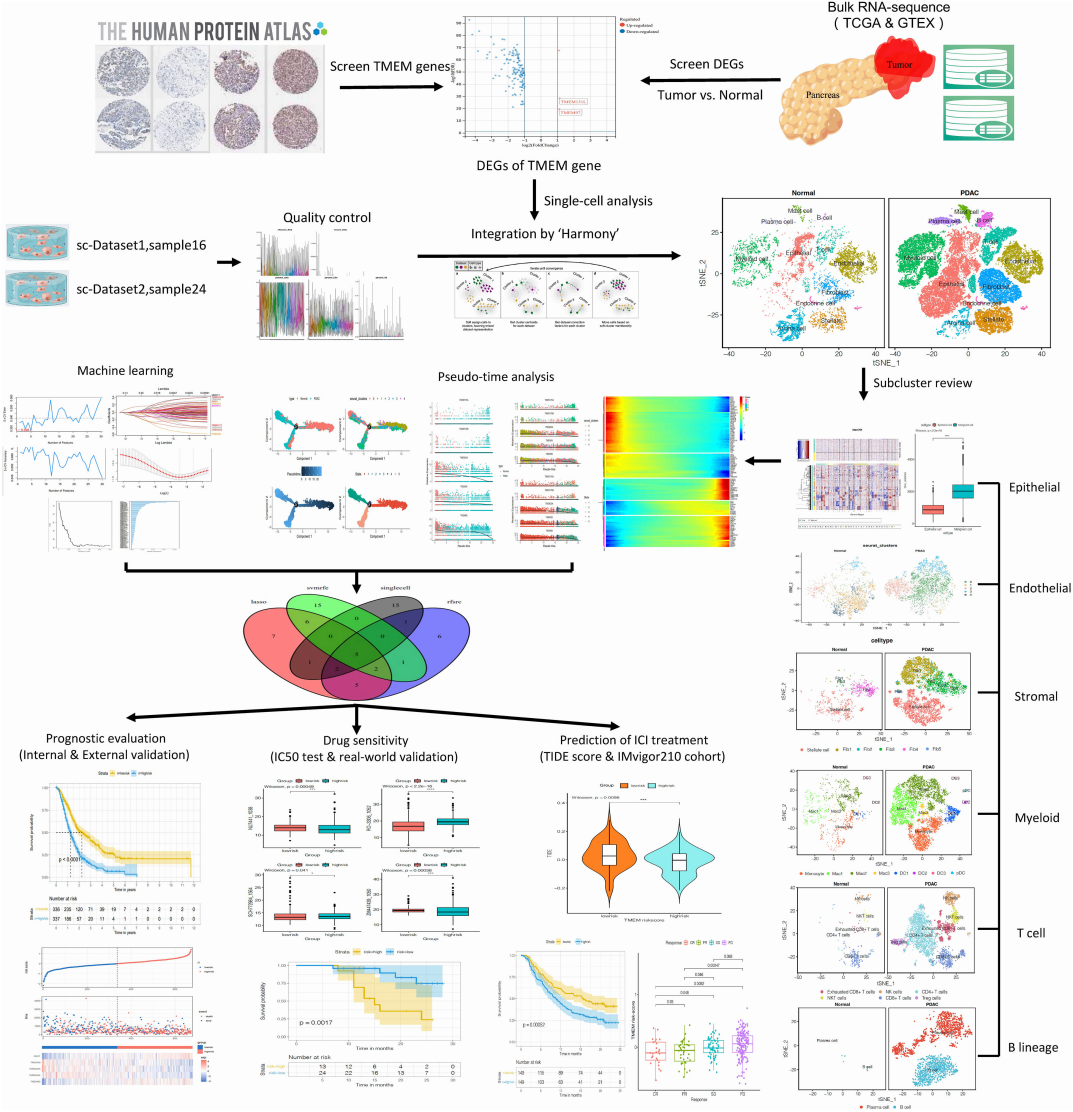
Schematic diagram for the total workflow of this study.

**Figure 1** illustrates the workflow of our research. Transcriptome data of pan-cancer and normal tissues from The Cancer Genome Atlas (TCGA) and The Genotype-Tissue Expression (GTEx) were downloaded using the UCSC Xena browser (https://xenabrowser.net/) (13). The RNA-sequence data of pancreatic cancer and normal pancreas were extracted for further analysis. We obtained microarray datasets, including GSE21501, GSE62452, and GSE57495 from the Gene Expression Omnibus (GEO) database, as well as bulk RNA sequence datasets from GSE 79668, PAAD-CA from ICGC, and our center’s tumor samples for analysis. Corresponding samples and clinical information for all included datasets were also prepared and organized. Single-cell transcriptome files of GSE155698 from GEO and CRA001160 from Genome Sequence Archive (GSA) were downloaded. The list of genes correlated with TMEM proteins was obtained from the Human Protein Atlas (HPA).

### 2.2 TMEM genes expression in bulk pancreatic tissues

The study compared the expression levels of TMEM genes in normal pancreas and PDAC tissues using the TCGA (tumor, N=150; normal, N=4) and GTEx (normal, N=70, filtered by RIN≥7) datasets, with transcripts per million (TPM) as the method of measurement. TMEM genes with an expression value of zero in more than half of tumor samples were filtered out, followed by identification of differentially expressed genes (DEGs) using the “Limma” package. DEGs were defined as genes with an absolute log2 fold change greater than 1, and multiple testing adjustice was performed using the Benjamini-Hochberg method.

### 2.3 Single−cell RNA−seq analysis

We conducted a single-cell analysis using the ‘Seurat’ R package to investigate two single-cell RNA-seq profiles of tumor and normal tissues. We used the following standards to exclude low-quality cells: 1) cells had fewer than 200 unique molecular identifiers (UMIs); 2) over 10% UMIs derived from mitochondrial genome; 3) less than 3% UMIs derived from ribosome genome; 4) over 1% UMIs derived from hemoglobin genome. 5) cells had average expression level of less than 3 genes. We then calculated the cell-cycle score using the ‘CellCycleScoring’ function, and removed the cell-cycle score and mitochondrial genome during the data scaling (14). We further selected G1 phase cells for analysis to eliminate the influence of the cell cycle (15). Integration and remove of batch effects of included single-cell RNA−seq datasets was applied by the R package “Harmony” (16). Dimensional reduction was performed using both UMAP and t-SNE method (17, 18). Identification of cell type for each cell cluster was performed using differentially expressed genes and marker genes reported in previous studies (19, 20). We investigated the expression of TMEM genes in clusters of different cell types, such as epithelial cells, fibroblast cells, T cells, and macrophages, within the tumor microenvironment. We also performed a pseudo-time analysis to explore the expression characteristics of both marker genes and TMEM genes in select cell types during the developmental trajectory of carcinogenesis. The pseudo-time processes were performed using the “monocle” package in R to reconstruct the trajectory.

### 2.4 Screening key TMEM gene through machine learning analysis

We choose five cohort (GSE21501, GSE62452, GSE57495, ICGC-PAAD-CA, and TCGA) as internal analytic datasets to evaluate prognostic TMEM genes. To identify clinical-relevant TMEM genes, we selected the intersection of TMEM genes obtained from the results of four machine learning algorithms: the Least Absolute Shrinkage and Selection Operator (LASSO) regression by the “glmnet” R package, Support Vector Machine-Recursive Feature Elimination (SVM-RFE) and random forest for survival, regression and classification (RF-SRC) (21–23) and single-cell analysis. Further, we validated the model established by those intersected genes and constructed the risks core signature named as “riskscore” by the coefficient factors obtained from multivariate cox analysis.

### 2.5 Prospective of TMEM gene riskscore in systematic therapy

To further elucidate the impact of TMEM riskscore, we classified patients into high-risk and low-risk groups and compared the clinical and pathological features between the two groups. Additionally, we explored the characteristics of the tumor microenvironment, including immune cell infiltration and immune checkpoint in both high-risk and low-risk groups of PDAC patients. We also conducted prognostic analyses in multiple datasets and constructed a prognostic risk prediction model based on TMEM riskscore using clinical and pathological data from TCGA.

We estimated the correlation between TMEM riskscore and the response of systematic therapy. TIDE algorithm was applied to assess the potential of immune checkpoint inhibitors therapy between the high and low-risk groups of TCGA patients (24). Then, we collected the transcriptome data and complete clinical information of the IMvigor210 cohorts to validate the potential of riskscore on immunotherapeutic efficacy (25). In addition, we screened and compared drug sensitivities based on transcriptome data of two groups through the R package “oncoPredict” (26).

### 2.6 Sample sources and clinical data

In order to corroborate the results of our investigation, we retrospectively analyzed data from 37 patients with pathologically confirmed pancreatic cancer who underwent radical resection, all of whom were consecutively treated at Peking University Third Hospital between January 2017 and January 2018. To be included in this validation cohort, patients had to meet the following criteria: (1) histologically confirmed pancreatic adenocarcinoma, (2) radical resection performed, (3) no in-hospital death after surgery, and (4) sufficient surgically resected tissue available for preservation in liquid nitrogen and further research. Patients were followed up every two months for the first six months, every six months for the next two years, and then annually thereafter. Image and tumor marker examinations were conducted routinely, and any new lesions detected by biopsy or imaging were considered indicative of tumor recurrence. Disease-free survival and overall survival were the primary endpoints of this study. The local research ethics committees approved this study with confirmation code M2016361, and informed consent was obtained from all patients in accordance with the committees’ regulations.

For validation of TMEM gene expression in protein level, we used tissue microarray (TMA) analysis which constructed by the tissue cores (4.00 mm in diameter) from the resection specimens of primary pancreatic cancer and paired adjacent normal tissue of 92 patients.

### 2.7 Bulk RNA-sequence and immunohistochemical staining

We conducted a concurrent validation of TMEM gene in mRNA level, using external RNA-sequence data from above-mentioned patients who underwent radical pancreatectomy for pancreatic ductal adenocarcinoma (PDAC). Detail information can be seen in **Supplementary File** and **Supplementary Table A-S8**. Kaplan-Meier survival analysis was employed to study the prognostic impact of riskscore in whole cohort and in every single dataset. External validation was conducted based on the data of GSE79668 and our center.

Immunohistochemistry was performed based on the tissue microarray. TMA slides were deparaffinized, rehydrated, and boiled in a pressure cooker filled with a sodium citrate buffer (pH 6.0) for antigen retrieval. After antigen retrieval, the slides were blocked with inhibitor (3% H2O2) for 30 min at 37°C. Immunohistochemical was performed using the rabbit polyclonal anti-TMEM92 antibody from atlas antibodies (HPA063009). Positivity for immunohistochemical staining was determined by the presence of brown particles within the cell membrane and cytoplasm. Color intensity was graded on a four-point scale based on the following categories: no pigment (0), light yellow (1), brownish-yellow (2), and dark brown (3). The percentage of stained cells within the microscope field of view was also graded on a four-point scale as follows: 0-25%, 26-50%, 51-75%, and > 75%, and scored as 0, 1, 2 and 3 points, respectively. The final score was calculated by multiplying the scores for color intensity and the percentage of stained cells. A final score of ≤ 6 points was considered indicative of low expression, while a score > 6 points was classified as high expression.

### 2.8 Cell culture

The pancreatic cancer cell lines CFPAC-1 was obtained from the Cell Resource Center, Peking Union Medical College (which is the headquarter of National Science & Technology Infrastructure–National BioMedical Cell-Line Resource, NSTI-BMCR) and cultured in Iscove’s Modified Eagle’s Medium (Hyclone Co., Logan, UT, USA) and supplemented with 10% FBS (Hyclone Co.) at 37°C in a humidified 5% CO2 air incubator.

TMEM92 siRNA#1 sequence were as follows: 5’−GCAGCCAAATGTGGTCTCA-3’. TMEM92 siRNA#2 sequence were as follows: 5’- CTGTCCGTCTTTTGCATCT-3’. TMEM92 siRNA#3 sequence were as follows: 5’- CCCAAAGGATTCAAATGCT-3’. A scrambled siRNA was used as negative control (si-NC). All above small interfering RNA were purchased from RiBoBio Co., Ltd. (Guangzhou, China). Cells were harvested 48 hours post-transfection and subjected to western blot analysis to evaluate the efficacy of the interference.

### 2.9 Western blot and quantitative real-time PCR (qRT-PCR)

Proteins were extracted from cells using RIPA lysis buffer with proteinase inhibitor. Then, proteins were separated by SDS-PAGE and transferred to polyvinylidene fluoride (PVDF) membranes. The membranes were blocked with 5% milk and 0.01% Tween-20 in tris-buffered saline (TBS; pH 7.6) and incubated with TMEM92 antibody diluted at 1:500 in TBS overnight at 4°C. GAPDH was used as an internal control. Protein quantification was performed in ImageJ.

RNA extraction and cDNA synthesis were performed using the TRIzol reagent (Corning co, USA) and PrimeScript RT Reagent Kit (Promega, Beijing, China), respectively. mRNA expression levels of TMEM92 were quantified using the SYBR Premix Ex Taq system (Promega, Beijing, China), with glyceraldehyde-3-phosphate dehydrogenase (GAPDH) expression levels serving as a normalization control. The relative levels of TMEM92 were determined using the comparative quantification cycle (Cq) method (2−ΔΔCq) based on three repeated measurements. The primers used in this study are listed below:

TMEM92 forward 5’-GCAGCCAAATGTGGTCTCATCC-3’
TMEM92 reverse 5’-GCAAAAGACGGACAGGATGACC-3’
GAPDH forward 5’-TGTGTCCGTCGTGGATCTGA-3’
GAPDH reverse 5’-CCTGCTTCACCACCTTCTTGA-3’.

### 2.10 Colony, migration and invasion assay

Cells were seeded into 6-well plates at 500 cells/well per dish in triplicate and cultured for 10 days. To evaluate colony formation, we fixed the colonies with a 4% paraformaldehyde solution for 15 minutes and stained them with crystal violet. The resulting colonies were then photographed and counted to determine their extent.

To assess the migration and invasion ability of pancreatic cancer cells, we utilized a transwell chamber. Specifically, we uniformly coated the upper surface of the chamber with 70 µL of Matrigel glue, sourced from BD Corporation (Franklin Lakes, NJ, USA). We then added 200 µL of a single cell suspension, containing 2 × 10 5 cells diluted with serum-free medium, to the upper chamber. Additionally, 500 µL of medium, containing 20% fetal bovine serum, was introduced to the lower chamber. Following 48 hours of incubation, we fixed the cells with 4% paraformaldehyde for 30 minutes and stained them with crystal violet for 30 minutes. Images were captured from five randomly selected fields under a microscope, and all experiments were performed in triplicate to ensure statistical validity.

### 2.11 Statistical analysis

The normality of the variables was tested by the Shapiro-Wilk normality test, unless otherwise specified. For the comparison of the two groups, the normal distribution variables were analyzed by the unpaired student t-test, and the non-normal distribution variables were analyzed by the Wilcoxon test. For comparisons of more than two groups, the Kruskal–Wallis test and the one-way ANOVA were used as non-parametric and parametric test methods, respectively. Spear-manand correlation analysis was used for analyzing the correlation coefficients. Two-sided Fisher exact tests were used to analyze contingency tables. And the Benjamini–Hochberg method was employed to convert P values to FDR. P value < 0.05 was used to determine the statistical significance of the difference. All of these analyses were performed in R 4.0.3 or SPSS version 26.0 software based on default parameters unless otherwise stated.

## 3 Results

### 3.1 Expression characteristics of TMEM genes in PDAC

There were 255 TMEM genes extracted for analysis between tumor and normal tissue after the preliminary filtration (**Supplementary Table A-S1**). And 128 TMEM genes were then identified as the significant differentially expressed gene (**Supplementary Table A-S2**). Only four TMEM genes were down-regulated and the rest 124 TMEM genes were indicated as up-regulated genes in PDAC (**Figure 1S**).

### 3.2 Single-cell transcriptomic analysis revealed heterogeneity of TMEM genes expression

**Figure 2:**
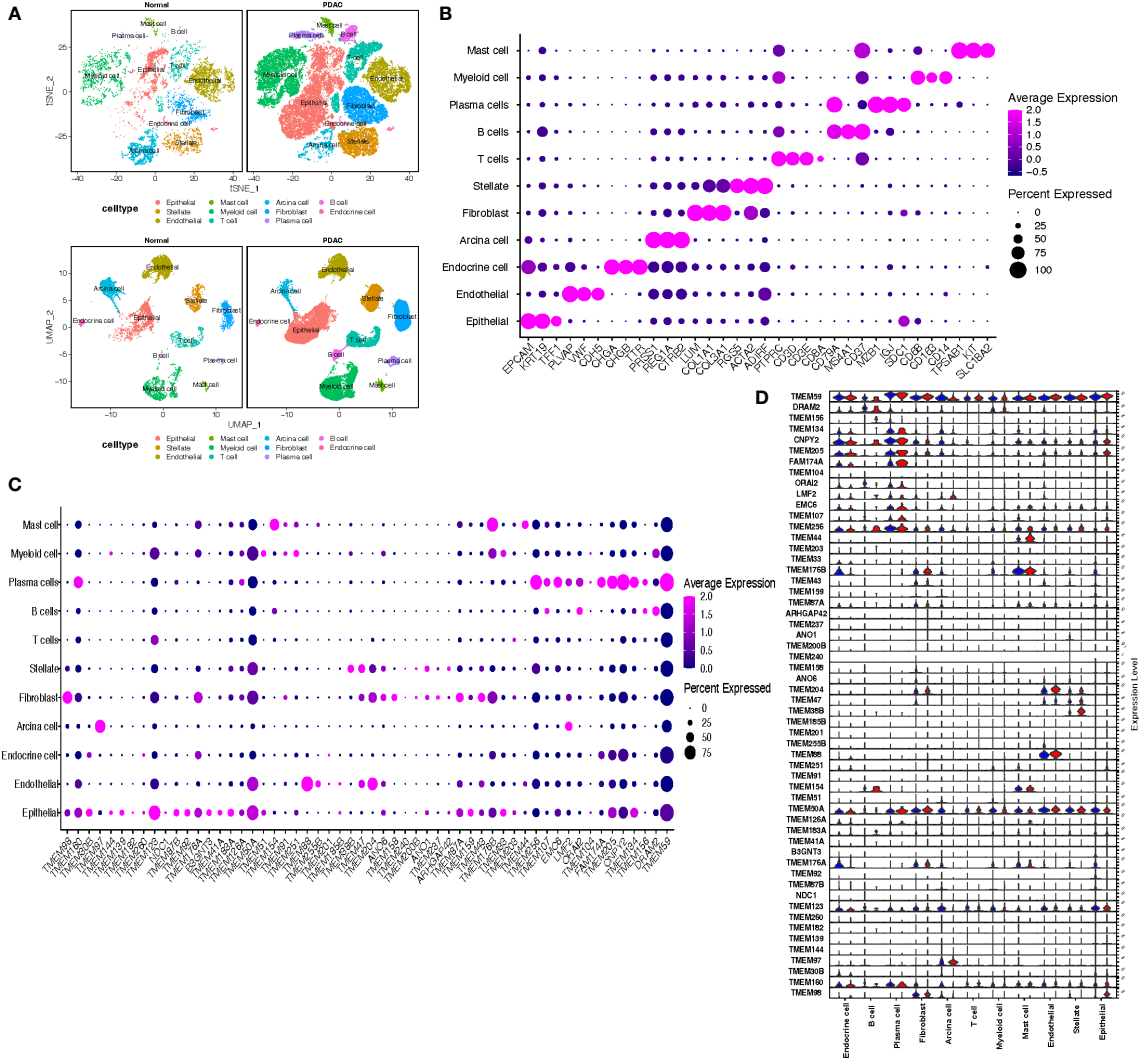
**(A)** t Stochastic neighbor Embedding (tSNE) and UMAP plots showing cell types for 53123 cells in normal pancreatic tissue and PDAC. **(B)** Dot plots showing the average expression distribution of marker genes in 11 cell types. **(C)** Dot plots showing the average expression distribution of TMEM genes in 11 cell types. **(D)** Violin plots showing the difference of TMEM gene expression in normal pancreatic tissue (blue violin) and PDAC (red violin) among 11 cell types.

**Figure 3:**
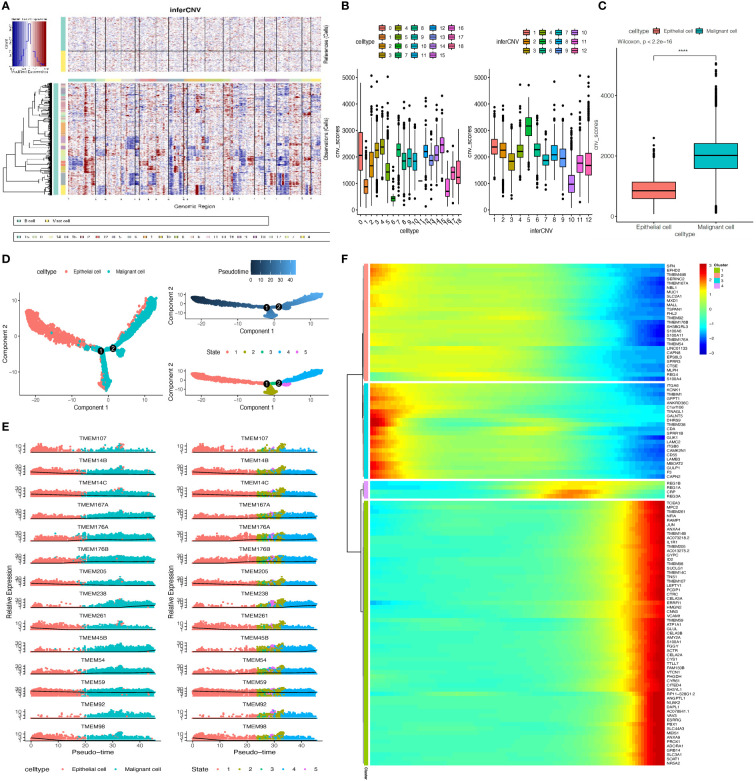
**(A)** Inference of copy-number variations (CNVs) for the 18 subgroups of epithelial cells refed by B cells and Mast cells. **(B)** Box plots depict the CNV score of all subgroups of epithelial cells. **(C)** Box plot showing the CNV score between normal epithelial cells and malignant epithelial cells, p-value calculated by Wilcoxon test. **(D)** The pseudo-time trajectory of epithelial cells. **(E)** Characteristics of TMEM gene expression in epithelial cells followed by the pseudo-time. **(F)** Heatmap showing dynamic expression of genes along the pseudo-time in epithelial cells trajectory. Rows of the heatmap represent genes that show dynamic changes along the pseudo-time, and these genes were clustered into four groups according to their expression pattern along the pseudo-time. (****P < 0.001).

**Figure 4:**
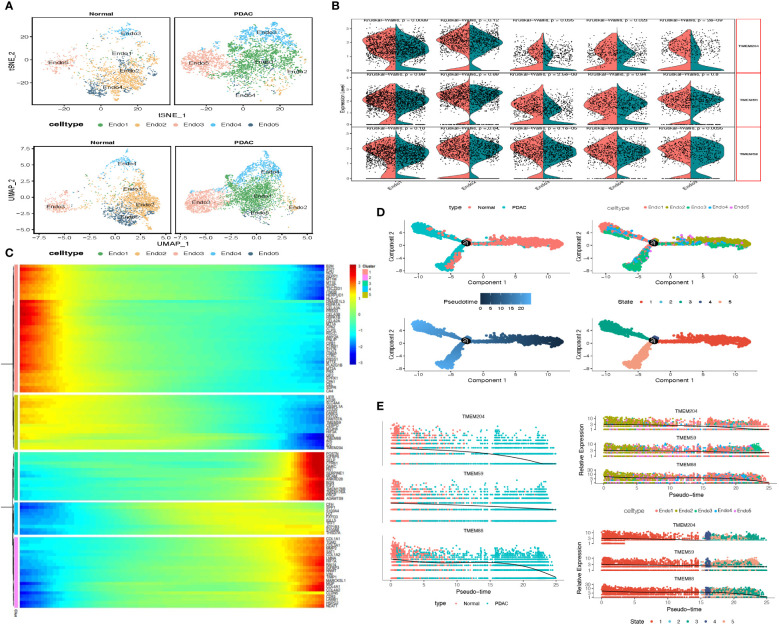
**(A)** tSNE and UMAP plot of the endothelial cells, color-coded for five clusters. **(B)** Split Violin plots demonstrated differences of TMEM gene expression among five subgroups of endothelial cells. **(C)** Heatmap showing dynamic expression of genes along the pseudo-time in endothelial cells trajectory. Rows of the heatmap represent genes that show dynamic changes along the pseudo-time, and these genes were clustered into five groups according to their expression pattern along the pseudo-time. **(D)** The pseudo-time trajectory of five endothelial cells subgroups. **(E)** Characteristics of TMEM gene expression in endothelial cells followed by the pseudo-time.

**Figure 5:**
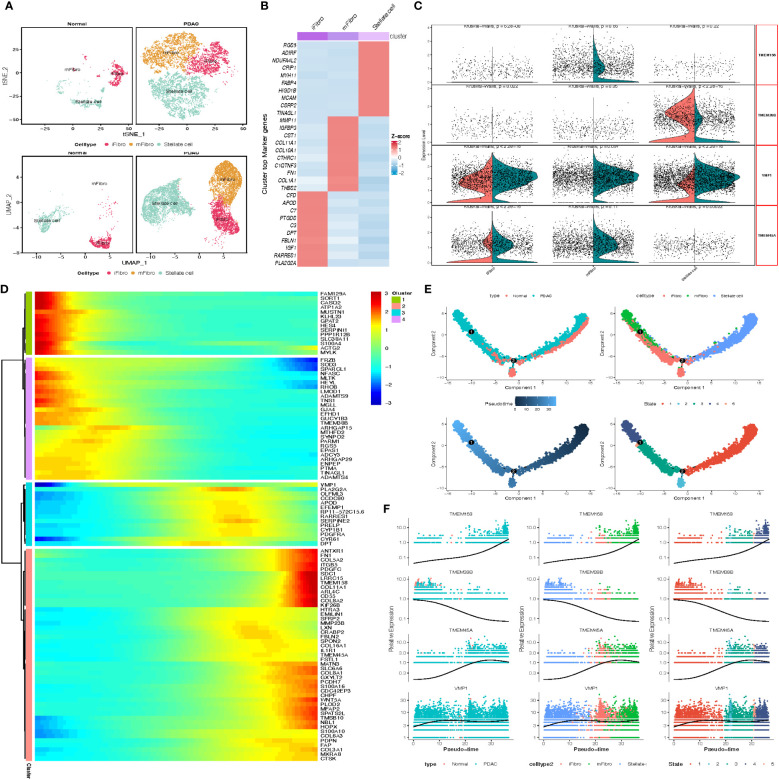
**(A)** tSNE and UMAP plot of the stromal cells, color-coded for three clusters. **(B)** Heatmap show the marker gene expression for 3 subclusters of stromal cells. **(C)** Split Violin plots demonstrated differences of TMEM gene expression among three subgroups of stromal cells. **(D)** Heatmap showing dynamic expression of genes along the pseudo-time in stromal cells trajectory. Rows of the heatmap represent genes that show dynamic changes along the pseudo-time, and these genes were clustered into four groups according to their expression pattern along the pseudo-time. **(E)** The pseudo-time trajectory of three stromal cells subgroups. **(F)** Characteristics of TMEM gene expression in stromal cells followed by the pseudo-time.

To assess the differences of TMEM gene expression among various infiltrating cells in the tumor microenvironment of PDAC, we conducted a single-cell analysis based on two single-cell RNA sequencing datasets (**Figure 2S**-**5S**). After the initial quality control, there were a total of 53123 cells retained from 14 normal pancreatic tissues and 40 pancreatic tumor tissues. Integration of each dataset and further dimension reduction was successfully achieved. The consequent graph-based analysis identified 11 cell clusters based on TSNE reduction and UMAP reduction (**Figure 2A**). These clusters were assigned to the following cell lineages through their own characterized differential genes compared with other clusters: epithelial cell (marked with EPCAM, KRT19, TFF1), endothelial cell (marked with PLVAP, VWF, CDH5), endocrine cell (marked with CHGA, CHGB, TTR), acinar cell (marked with PRSS1, REG1A), stellate cell (marked with RGS5, ACTA2, ADIRF), fibroblast (marked with LUM, COL1A1, COL3A1), myeloid cell (marked with CD68, CD163, CD14), T cell (marked with CD3D, CD3E, CD8A), B cell (marked with CD79A, MS4A1, CD37), plasma cell (marked with MZB1, IGJ, SDC1) and mast cell (marked with TPSAB1, KIT, SLC18A2) (**Figure 2B**; **Supplementary Table A-S3**).

The differential expression TMEM genes explored in the bulk RNA-sequence analysis were firstly applied for global assessment in pancreatic tissues. The outcomes denoted there is significant heterogeneity observed in TMEM genes expression, besides, among 11 different cell types, epithelial cells were characterized by TMEM30B, TMEM139, TMEM123, TMEM87B, TMEM92, B3GNT3, TMEM41A, TMEM183A, TMEM159; endothelial cells were characterized by TMEM88, TMEM255B, TMEM204; acinar cells were characterized by TMEM97; stellate cells were characterized by TMEM38B, TMEM47 and ANO1; fibroblast were characterized by TMEM97, TMEM158, TMEM237 and TMEM43; T cells were characterized by TMEM203; B cells were characterized by TMEM107,DRAM2, TMEM156 and ORAI2; plasma cells were characterized by TMEM160, TMEM256, EMC6, FAM174A, TMEM205,CNPY2,TMEM134 and TMEM59; myeloid cells were characterized by TMEM51, TMEM251 and TMEM33; and mast cells were characterized by TMEM154, TMEM176B and TMEM44 (**Figure 2C**). Then, the TMEM genes with remarkable different expression between tumor and normal tissue were also analyzed based on above 11 cell lineages (**Figure 2D**). In order to reduce the deviation caused by normal pancreatic ductal cells in tumor tissues, we conducted the inference of copy-number variations in epithelial cells to distinguish the malignant and normal cells based on expression profiles (**Figures 3A, B**). Referring to immune cells, there were 1691 epithelial cells reviewed with significant lower CNVs, considered as normal ductal cells (normal vs. malignant cells: 883.6 ± 389.6 vs. 2011.9 ± 631.0, P<0.01, **Figure 3C**; **Supplementary Table A-S4**). Subsequently, to select the tumor-associated TMEM genes, we conducted differential genes analysis and pseudo-time analysis (**Figure 3D**). In epithelial cells, 13 TMEM genes present as DEGs (logFC>0.58, P<0.01). Further pseudo-time analysis revealed TMEM238, TMEM176A, TMEM176B, TMEM45B, TMEM54, TMEM92, and TMEM167A were present with an elevated tendency of expression in malignant cells over the pseudo-time. Meanwhile, TMEM14C, TMEM107, TMEM205, TMEM14B, TMEM261, and TMEM98 were present with a decreased tendency of expression over the pseudo-time (**Figure 3E**). In addition, the outcomes of enrichment analysis of each cluster in the pseudo-time gene expression heatmap demonstrated those TMEM genes significantly correlated to abnormally increased metabolism, tube development, pancreatic secretion, negative regulation of dendritic cells and extracellular matrix organization (**Figure 3F**; **Supplementary Table B**).

To learn the TMEM gene expression in endothelial cells, we firstly conducted dimension reduction for total endothelial cells to identify new clusters. Finally, 6265 endothelial cells were divided into 5 prominent subgroups (Endo1-Endo5, **Figure 4A**). We observed that 3 endothelial subgroups: Endo1 (97.6%), Endo3 (80.5%) and Endo4 (83.3%), were mainly involved in tumor tissue, and the normal tissue consisted of almost entirely Endo2 (91.8%) and a large proportion of Endo5 (80.8%) endothelial cells. Subsequently, we performed an investigation of DEGs among each subgroup and consequent enrichment analysis of the top 30 DEGs of each subgroup (**Supplementary Table A-S5**). Endo1 was present with high expression of COL4A1, COL15A1 and VWA1, characterized by enrichment of extracellular matrix organization and external encapsulating structure organization. Endo2 was shown with high expression of CLPS, CA4 and CTRB1, characterized by enrichment of digestion, water-soluble vitamin metabolic process, and intestinal cholesterol absorption. Endo3 was shown with high expression of DARC, CPE and POSTN, enriched in cellular extravasation, response to interleukin-1, and platelet degranulation. Endo4 were present with high expression of FABP4, IGFBP3 and GJA4, characterized by enrichment of receptor-mediated endocytosis endothelium development and integrin binding. Endo5 was shown with high expression of MT1A, STC and HSPA1A, enriched in protein folding chaperone, kinase inhibitor activity and negative regulation of growth. Furthermore, there were 5 TMEM genes identified as DEGs, and in pseudo-time analysis, only TMEM204, TMEM59, and TMEM88 were observed with an evident downward trend of expression (**Figure 4B**). Complied with this trend, the heatmap depicts that Endo5-cluster genes have similar expression trends followed by pseudo-time (**Figures 4C–E**). Our enrichment analysis shows that these genes are mainly involved in ion transport and regulation of metabolism (**Supplementary Table B**).

We next investigated the two types of stromal cells, the stellate cells and fibroblasts. We obtained 4081 stellate cells and 5843 fibroblasts that were clustered into 3 subgroups, including 1 stellate cell cluster and 2 fibroblast clusters (inflammatory fibroblast, iFibro; myofibroblast, mFibro, **Figure 5A**). According to their top 30 DEGs and further enrichment analysis, we performed designment of each fibroblast subcluster (**Figure 5B**). Subcluster mFibro accounted for the majority of the fibroblast populations in tumor tissue and expressed a high level of COL1A1, MMP11, FN1 and POSTN, which were associated with collagen secretion and extracellular matrix modeling. Subcluster iFibro was present with a high level of FBLN1, IGF1, CTSC and markers of the complement system (C3, C7, C1S, C1R), and designated as inflammatory fibroblast. Subsequently, TMEM genes expression of stromal cells in normal and tumor tissues was compared, and TMEM158, TMEM38B, VMP1 and TMEM45A were recognized as DEGs (**Figure 5C**). Then, further analysis revealed the expression of TMEM158 and TMEM45A was depicted with synchronized increase followed by pseudo-time, and TMEM38B was reversed (**Figures 5D–F**). Besides, the heatmap and GO analysis demonstrated both the elevated expression of TMEM158 and TMEM45A was highly associated with ECM organization and structural constituent. However, the depressed level of TMEM38B might be correlated with dysfunction of calcium ion signaling in PDAC (**Supplementary Table B**).

**Figure 6:**
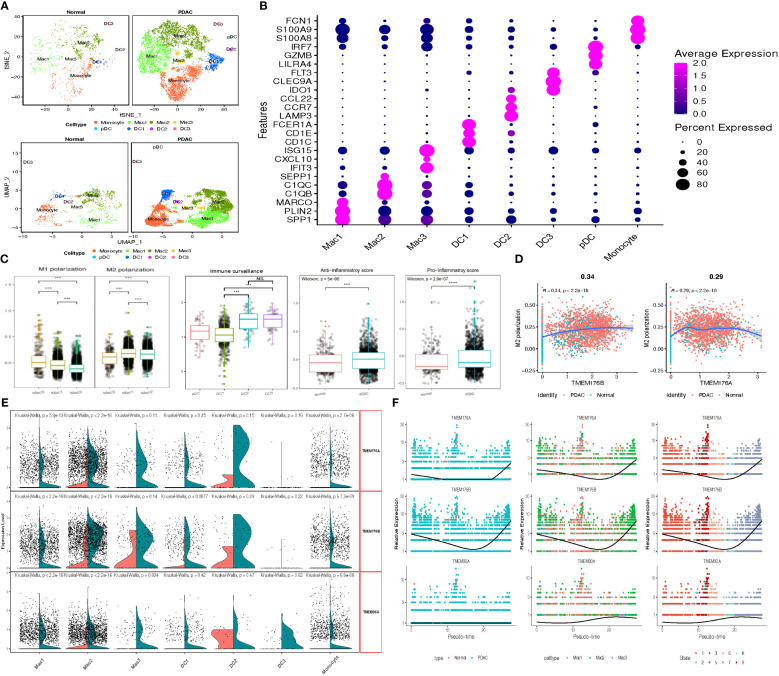
**(A)** tSNE and UMAP plot of the myeloid cells, color-coded for eight clusters. **(B)** Dot plot reveal the average expression of marker gene for 8 subclusters of myeloid cells. **(C)** Box plots demonstrated M1 & M2 polarization score in macrophage, immune surveillance score in DCs, Anti-&Pro-inflammatory score in normal and tumor-infiltrating monocytes. **(D)** Scatter plots depicted the significant correlation between M1 & M2 polarization score and TMEM176 with Spear-manand test. **(E)** Split Violin plots demonstrated differences of TMEM gene expression among seven subgroups of myeloid cells. **(F)** Characteristics of TMEM gene expression in three subgroups of macrophage followed by the pseudo-time. (***P = 0.001; ****P < 0.0001; NS, not significant).

TMEM gene expression of myeloid cells was then estimated based on subcluster identification. The reduction plot exhibited 7 subclusters, including 3 macrophage subclusters, 3 dendric cell subclusters and 1 monocyte subcluster (**Figure 6A**). Designation of each subcluster was conducted using their marker genes, Mac1 (SPP1, PLIN2, MARCO), Mac2 (C1QB, C1QC, SEPP1), Mac3 (ISG15, CXCL10, IFIT3), DC1 (CD1C, CD1E, FCER1A), DC2 (LAMP3, CCR7, CCL22), DC3 (IDO1, CLEC9A, FLT3), pDC (LILR4, GZMB, IRF7), Monocyte (S100A8, S100A9, FCN1) (**Figure 6B**; **Supplementary Table A-S6**). The distribution of each myeloid subcluster cells in normal and tumor tissue was variable. Tumor tissues were processed with larger percentage of Mac1, Mac3, DC1 and DC2 (tumor vs. normal: 31.6% vs.19.5%, 2.1% vs. 0.86%, 7.6% vs. 4.3%, 0.9% vs.0.3%), instead, normal tissues were observed with relatively more DC2, Mac2 and monocytes dwelled in. Considering the heterogeneity among these subpopulations of cells, the M1 and M2 polarization score of macrophages, the anti-inflammatory and pro-inflammatory score of monocytes, and the immune-surveillance and immune-escape score of DC cells were calculated through the ‘AddModuleScore’ function for pathophysiological estimation (**Figure 6C**; **Supplementary Table A-S9**). Moreover, we identified TMEM176B as DEGs (logFC>0.58, P<0.01) and other TMEM genes (TMEM176A and TMEM30A) with closely differential expression (0.25<logFC<0.58). Additional outcomes revealed the expression of TMEM176A and TMEM176B was almost parallel followed by pseudo-time, and evidently elevated in tumor-infiltrating Mac2 and DC1 (logFC>1, P<0.01). To be noticed, subcluster Mac2 and DC1 earned the lowest scores in M1 polarization score and immune surveillance score respectively, which indicated TMEM176A and TMEM176B might participate in the immune processes such as immune phagocytosis of macrophage and cell antigen presentation of DC cells (**Figures 6D–F**).

We also analyzed TMEM gene expression of T cells and NK cells within the tumor microenvironment of pancreatic cancer (**Figure 6S**-**9S**). The DEG analysis between tumor and normal tissues showed the expression of TMEM123 was significantly increased in tumor-infiltrating T cells, which was consistent with the analysis of bulk transcriptomic outcomes. We compared the TMEM123 expression between tumor and normal cells in 3 subgroups of T cells, in which differential significance was observed in the T memory and effect memory cells, indicating high expression of TMEM123 of tumor-infiltrating CD8+ T cells might be associated with the immune response to malignancy. In addition, TMEM66 was revealed with relatively lower expression in tumor-infiltrating CD8+ T cells. The next pseudo-time analysis demonstrated the expression level of TMEM66 combined with the genes enriched in regulation of the immune response and apoptotic process was parallelly depressed, however, elevated expression of TMEM123 might be associated with an enhanced cytotoxic function of CD8+ T cells.

Previous research reported abundant infiltration of activated B cells located in tertiary lymphoid structures might be a prognostic indicator in many cancers (27). Therefore, we briefly explored the changes of TMEM gene expression in B-lineage lymphocytes in the tumor microenvironment during activation of B cells (**Supplementary Table A-S7**). Preliminary outcomes from DEGs analysis identified 8 differential expression TMEM genes (logFC>1, p<0.01), and further results of pseudo-time analysis showed increased expression of TMEM208, TMEM59 and TMEM258, which are enriched in protein processing and binding, might be probably associated with transformation from B cell to plasma cells. However, considering that above genes are overwhelmingly expressed in plasma cells, we speculate that the increase of the first activated B cell infiltration in the tumor microenvironment leads to the up-regulation of the corresponding TMEM gene expression (**Figure 10S**-**12S**).

Taken together, these results suggest that the differential expression of TMEM genes is closely related to the infiltration of specific cells in the tumor microenvironment and the alteration of cell function during tumor development, and may finally contribute to the change of tumor phenotype and heterogeneity. Therefore, we screened 24 TMEM genes for subsequent analysis based on the above research.

### 3.3 Identification of the Key TMEM genes

**Figure 7:**
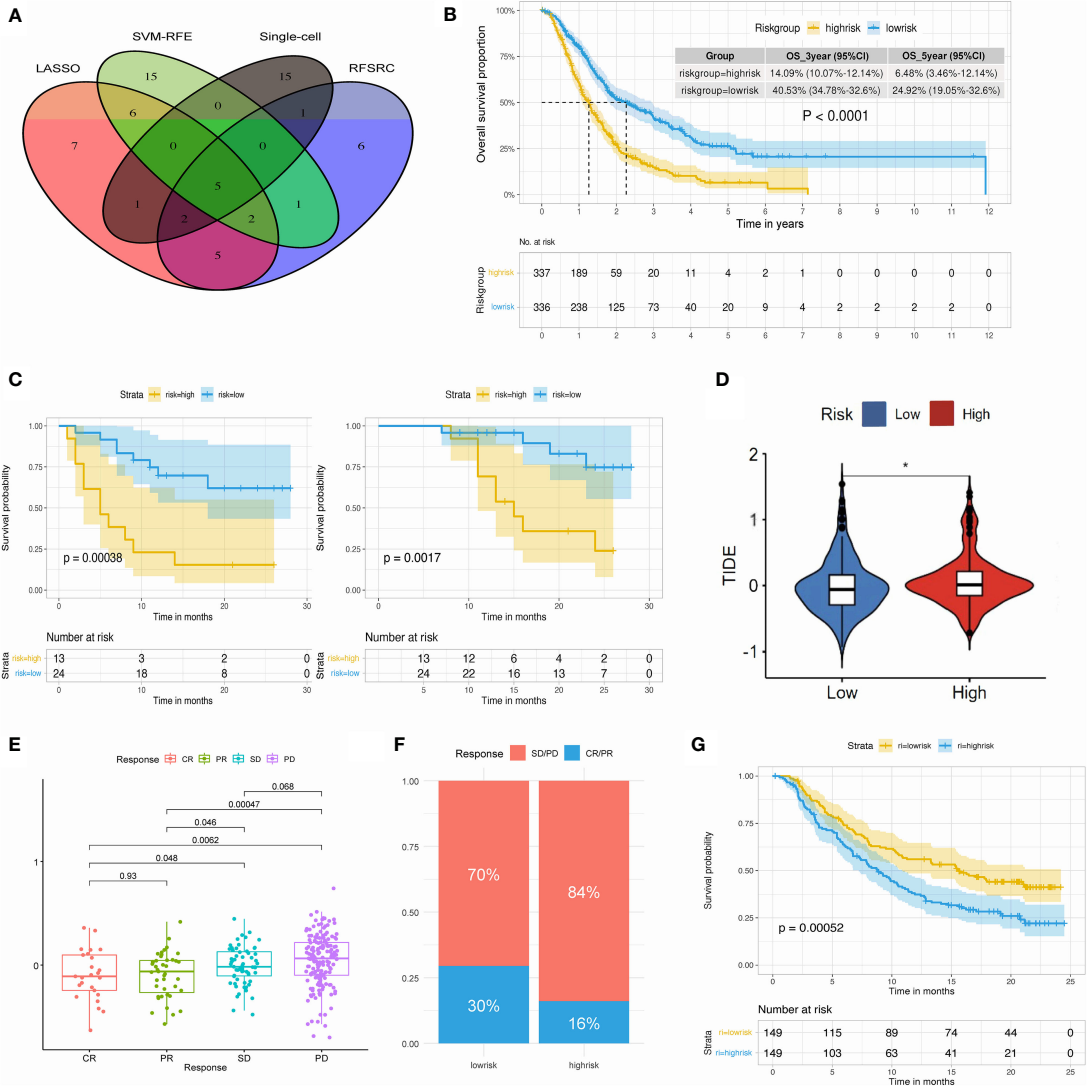
**(A)** Venn plot select the key TMEM genes. **(B)** K-M plot of 673 PDAC patients revealed distinct overall survival in high- and low-risk group. **(C)** Kaplan-Meier plot of 37 PDAC patients revealed distinct DFS (left) and OS (right) in high- and low-risk group. **(D)** Violin plot of TIDE score in high- and low-risk group. **(E)** Box plot showing the TMEM riskscore in patients within different immunotherapy response in the IMvigor210 cohort. **(F)** Bar plot showing the percentage of immunotherapy response in high- and low-risk group. **(G)** Kaplan-Meier plot showed the significant difference of OS between high- and low-risk group in the post-immunotherapy cohort. (*P < 0.05).

In order to detect the key TMEM genes, we first conducted the survival analysis based on 5 pooled datasets (GSE21501, GSE62452, GSE57495, TCGA-PAAD, ICGC-PACA). The outcomes obtained from the univariate analysis revealed 16 TMEM genes identified as favorable prognostic factors and 18 hazard prognostic factors for overall survival. In addition, three machine learning approaches: LASSO, SVM-RFE and RF-SRC, successfully achieved 28, 29, and 20 prognostic TMEM genes, respectively (**Figure 13S**). Furthermore, combined with the results arrived from single-cell transcriptomic analysis and bulk RNA-sequence analysis, we intersected those genes and finally obtained 5 key TMEM genes (ANO1, TMEM59, TMEM204, TMEM205, TMEM92), which were depicted in **Figure 7A**.

### 3.4 TMEM riskscore predict prognosis and response to systematic therapy

The establishment of TMEM genes signature risk score based on the above 5 TMEM genes was conducted, then we stratified the risk score into two groups (high-risk & low-risk) with the median value and explored the impact of risk score on clinical prognosis and systematic therapy (**Figure 14S**-**15S**).

In terms of clinical pathology data, our observations indicate that pancreatic cancer patients who were over 65 years of age, had a G3-4 tumor grade, and T3-4 tumor stage exhibited higher TMEM risk scores (**Figure 16S**). In the context of immune cell infiltration in the tumor microenvironment, we employed the CIBERSORT algorithm to compare the differential infiltration of cells between pancreatic cancer patients with high and low risk scores (**Figure 17S**). Our findings indicate that patients with low-risk scores exhibit increased infiltration of CD8+ T cells in the tumor microenvironment, whereas patients with high analysis scores exhibit elevated infiltration of macrophages. Five key transmembrane protein genes are also related to the infiltration of PDAC immune cells (**Figure 18S**). Moreover, we observed significant disparities in the expression of various immune checkpoint genes between the two cohorts (**Figure 19S**).

Kaplan-Meier survival analysis depicted a remarkable discrepancy of 3-year survival rate, 5-year survival rate and overall survival between high and low risk group (**Figure 7B**; **Figures 20S**-**22S**). Independent analysis based on two external datasets (GSE79668 and our datasets) also verified the prognostic impact of TMEM genes riskscore (**Figure 23S**; **Figure 7C**; **Supplementary Table A-S8**).

In the following evaluation of TMEM gene riskscore on immune therapy, the TIDE score was quite different in two groups (**Figure 7D**). Low-risk group was observed with the low TIDE score, which indicated patients in low-risk group might benefit from the ICIs treatment (**Figure 24S**). Additional analysis from IMvigor210 cohorts concurred with this finding and pointed out a valuable potential of the TMEM riskscore in the prediction of treatment response and post-treatment survival (**Figures 7E–G**).

Moreover, we conducted the prediction of IC50 value for common drugs used in pancreatic cancer to compare their treatment sensitivities (**Figure 25S**-**26S**) The results suggest that patients in the high-risk group might benefit more from the gemcitabine and paclitaxel, while the low-risk group might benefit from the oxaliplatin and irinotecan. Therefore, we performed a subgroup analysis of patients with adjuvant chemotherapy in our center, and the outcomes showed distinct OS and DFS intervals of patients who received gemcitabine-based chemotherapy, indicating that TMEM riskscore is sensitive for prediction of post-operative survival for patients with delivery of adjuvant chemotherapy (**Figure 27S**).

### 3.5 Validation of high TMEM92 expression and its prognostic impact in PDAC

**Figure 8:**
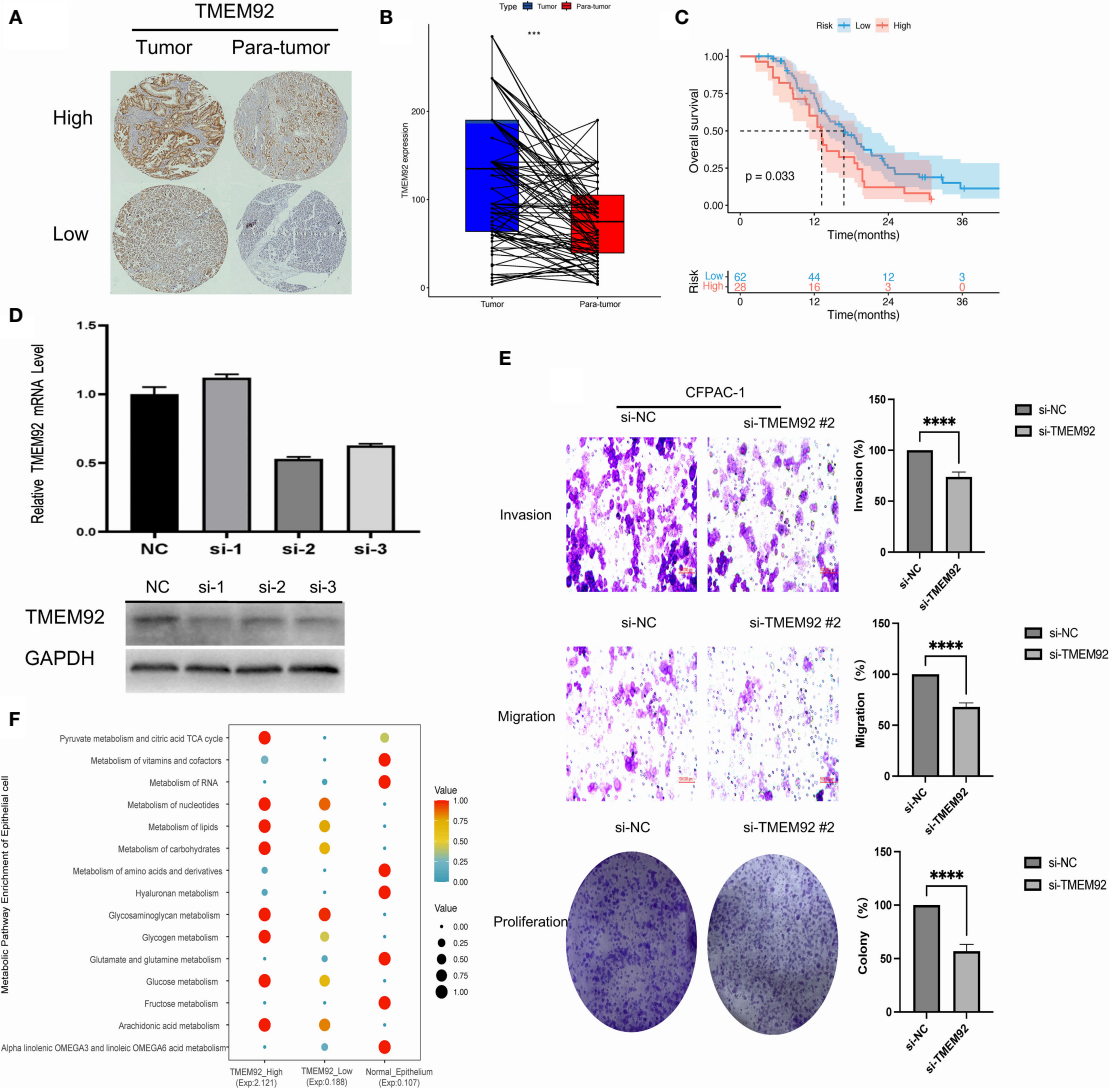
**(A)** The protein expression of TMEM92 in immunohistochemical images of tumor (left) and para-tumor (right) groups. **(B)** Pairwise analysis revealed remarkably elevated expression of TMEM92 in PDAC. **(C)** Survival analysis of TMEM92 protein expression in patients with PDAC. **(D)** CFPAC-1 cell was treated using siRNA to inhibit TMEM92 expression. Downregulated TMEM92 expression was examined by Western blot and qRT-PCR analyses. **(E)** Representative photographs showed distinct migration, invasion and proliferation of downregulated TMEM92 in CFPAC-1 cell lines. Data shown represent the mean ± SD. **(F)** Dot plot showed the difference of metabolism in epithelial cells with high and low expression of TMEM92 in single-cell analysis. (***P = 0.001; ****P < 0.0001).

46 paired samples of pancreatic cancer and its adjacent normal tissue were tested in the TMA slides. Immunohistochemistry assays showed that TMEM92 was mainly localized on the cell membrane and cytoplasm (**Figure 8A**; **Figure 28S**). Subsequent analysis of immunohistochemical stanning reveal the protein expression of TMEM92 was relatively higher in PDAC tissues (**Figure 8B**). Significant upregulation of TMEM92 was observed in 25 cases of pancreatic cancer tissue, and survival analysis revealed a strong correlation between elevated TMEM92 expression and poor prognosis (**Figure 8C**).

### 3.6 Knockdown of TMEM92 suppressed proliferation, and invasion in pancreatic cancer cell lines

Knockdown of TMEM92 was achieved *via* siRNA technique, which was validated through qRT-PCR and Western blot assays in the same cell line. By performing transient transfection experiments using three different siRNAs on the CFPAC-1 cell line, we identified the siRNA with the most desirable knockdown effect. Among the three siRNAs tested, si-2 exhibited the most significant knockdown effect and was subsequently selected for further knockdown experiments (**Figure 8D**). Our findings reveal that silencing TMEM92 expression leads to a significant reduction in the proliferative function of CFPAC-1 cells (**Figure 8E**). Migration and invasion assays also demonstrate that cells with downregulated TMEM92 exhibit a marked decrease in their migratory and invasive capabilities. These results implicate TMEM92 as an essential regulator of pancreatic cancer cell proliferation, migration, and invasion. Moreover, our single-cell analysis reveals a striking enrichment of lipid metabolism and glucose metabolism in malignant epithelial cells with heightened TMEM92 expression (**Figure 8F**). Such metabolic alterations may underlie the aggressive biological behavior of pancreatic ductal adenocarcinoma (PDAC). In summary, our findings provide critical insights into the role of TMEM92 in PDAC, highlighting its potential as a therapeutic target for this devastating disease.

## 4 Discussion

Transmembrane proteins are critical for the transportation of substances and biological signals in various physiological and pathological processes between cells (28). Although their simple functions are well-established, the widespread and diverse expression across different tissues and cell types suggests that their impact could be substantial and intricate (29). In our study, we discovered a significant number of TMEM genes with abnormal expression in PDAC. Notably, these abnormalities were observed not only in the malignant cells themselves, but also in several types of cells present in the tumor microenvironment. Therefore, in combination with single-cell transcriptome sequencing, analyzing the expression characteristics of TMEM protein genes can lead to more advanced strategies for understanding the potential functions and effects of these proteins in the tumor microenvironment (30, 31).

In the single-cell analysis, we observed abnormal expression of 13 TMEM genes in malignant epithelial cells, including genes that co-regulate cellular metabolism and immune signal transduction. This is consistent with the previous studies on metabolic-pathway-based subtyping of pancreatic cancer which reported distinct lipid and glycolysis metabolism, however, the regulatory genes of tumor metabolism still remain unclear (32). These TMEM genes may probably participate in the regulation of metabolism through some pathways. Previous research demonstrated TMEM176A may participate in tumor invasion through the EMT process (33). In our study, we found up-regulated expression of TMEM176A and TMEM176B both in malignant cells and myeloid cells, which might negatively affect the differentiation of DC cells leading to the potential imbalance of antigen presentation and promoting tumor immune escape (34).

In the tumor microenvironment, the up-regulation of three TMEM genes (TMEM204, TMEM88, and TMEM59) in endothelial cells caught our attention. However, subsequent survival analysis revealed that their elevated expression was actually a favorable factor for prognosis. We propose that this contradiction may be attributed to the dynamic changes in the tumor stroma (35). High expression of these three TMEM genes was found to be correlated with angiogenesis and ECM organization in endothelial cells. The occurrence and progression of tumors promote vascular infiltration in the microenvironment, which is consistent with the increased expression of these genes observed in pseudo-time analysis. However, as the tumor advances, the formation of a dense extracellular matrix and hypoxia can lead to a hypovascular microenvironment, resulting in relatively lower levels of gene expression compared to the initial phase of tumor (36). Furthermore, the identification of subclusters of tumor-infiltrating endothelial cells has piqued our interest in discerning potential pro-tumor and anti-tumor endothelial cells (37). Pro-tumor endothelial cells may participate in the development of malignant cells and remodeling of the tumor microenvironment, while anti-tumor endothelial cells may be involved in angiogenesis and immune activation. Therefore, our future efforts will focus on identifying pro- and anti-tumor endothelial cells using weighted gene co-expression network analysis and exploring potential therapeutic targets. This could potentially yield more meaningful results in identifying TMEM gene expression in different subtypes of endothelial cells.

We delved deeper into the expression characteristics of TMEM genes in heterogeneous stromal cells in PDAC, and investigated the changes in TMEM gene expression during the transformation of stellate cells into tumor-infiltrating fibroblasts. ANO1 is a calcium activated chloride channel fast membrane protein (38). Current studies have shown that ANO1 is closely related to the development, invasion and metastasis of several malignancy (39, 40). Our findings confirmed that high expression of ANO1 is a factor in unfavorable prognosis, and also revealed that the increased level of ANO1 was closely related to ECM remodeling and influenced by denser infiltration of myofibroblasts. Furthermore, we suggest that the dynamic change of ANO1, TMEM38B, TMEM158, and TMEM45A may indicate stellate cell activation and trigger tumor stromal remodeling. However, the mechanism underlying these changes remains unknown and requires verification *in vitro (* 41, 42).

We conducted an investigation of the alteration of TMEM genes in tumor-infiltrating immune cells, including macrophages, DC cells, T cells, and B lineage cells, in pseudo-time. Previous studies have reported that abundant infiltration of SPP1+ macrophages is frequently observed in the tumor microenvironment and is significantly associated with poor prognosis (43, 44). Our findings confirmed the abundant infiltration of C1QC+ and SPP1+ macrophages, which is consistent with these previous studies. Using the M1 and M2 polarization score to quantify the immune function among different subtypes of macrophages, we suggest that tumor-infiltrating macrophages may promote the construction of an immunosuppressive tumor microenvironment by regulating the expression of TMEM176 (43). Furthermore, we observed that TMEM176 is also involved in immune regulation, as it is significantly highly expressed in DC1 cells, which are associated with impoverished immune surveillance. TMEM176 has been demonstrated to be strongly correlated with the infiltration of immune cells in tumors (45, 46). TMEM176B inhibits NLRP3 inflammasome activation to regulate adaptive and innate antitumor responses, suggesting that TMEM176 may be a potential immunotherapy target for pancreatic cancer (47). Additionally, numerous studies have shown that TMEM123 and TMEM66 in T cells, and TMEM208, TMEM59, and TMEM258 in B cells, are closely related to inflammatory response and participate in tumor immune response, which is consistent with our findings (48–50).

In order to gain a further understanding of the impact of transmembrane protein gene expression on pancreatic cancer prognosis, we conducted a comprehensive bioinformatics analysis. Our study has identified 5 key transmembrane protein genes associated with pancreatic cancer prognosis, and we have constructed a reliable predictive model for assessing the prognostic impact of TMEM genes. Based on this model, we found that patients with high and low TMEM risk scores had significant differences in clinical pathology and immune infiltration. Specifically, patients with high TMEM risk scores were associated with an immunosuppressive tumor microenvironment, indicating a potential mechanism for their poor prognosis. Our findings suggest that these five key TMEM genes have significant clinical implications for pancreatic cancer prognosis and could potentially be utilized as prognostic biomarkers in clinical practice. Single-cell analysis revealed that the expression of these five genes was heterogeneous across different cell types. Based on the expression characteristics of these genes, we found that the TMEM92 gene was highly expressed in malignant epithelial cells of pancreatic cancer and was significantly associated with an immunosuppressive tumor microenvironment. To further investigate the clinical significance of TMEM92 in pancreatic cancer, we performed immunohistochemical analysis and found that patients with high TMEM92 expression had poor prognosis. Furthermore, functional analysis of cell lines demonstrated that interference with TMEM92 expression by siRNA significantly down-regulated the clone formation and cell invasion abilities of CFPAC-1 cell line. These findings suggest that the TMEM92 gene may be associated with malignant features of pancreatic cancer and its potential mechanism may involve the regulation of substances such as glycogen, cholesterol, and lipids to regulate malignant tumor epithelial cells.

Despite the promising results of our study, we acknowledge that our findings have some limitations. While we identified five key TMEM genes associated with pancreatic cancer prognosis, the predictive value of our model, with a C-index of 0.62, was relatively inferior to that of other models reported in previous studies. studies (51, 52). Therefore, we believe that future research should focus on not only identifying prognostic TMEM genes, but also on understanding the biological behavior of tumors through TMEM gene alterations in the microenvironment, which may lead to the discovery of novel therapeutic targets in the future.

Overall, this study for the first time revealed that TMEM genes were dysregulated in PDAC samples by analyzing single-cell and bulk RNA-sequence. Single-cell and bulk-RNA sequence facilitated the exploration of differential expression and dynamic alteration of TMEM genes. We investigated the characteristics of TMEM gene expression in several types of cells embedded in the tumor. Our research reported that 24 TMEM genes with remarkable differential expression might attribute to the remodeling of the tumor microenvironment and immune response through single-cell analysis. In addition, through the machine learning algorithm, 5 key TMEM genes were identified. We then evaluated the prognostic impact of TMEM gene expression and therapeutic response of systematic therapy in patients with different risk stratification. These results uncovered the pattern of TMEM gene expression in PDAC and its effect on clinical application, laying a novel theoretical target for PDAC treatment.

## Data availability statement

The original contributions presented in the study are included in the article/**Supplementary Material**. Further inquiries can be directed to the corresponding author.

## Ethics statement

The studies involving human participants were reviewed and approved by the Ethics Committee of Peking University Third Hospital. The patients/participants provided their written informed consent to participate in this study.

## Author contributions

SR: study design, analysis, and interpretation of data, drafting the article, final approval; AS: study design, analysis, and interpretation of data, drafting the article, final approval; XG: acquisition of data, drafting the article, revising the article, final approval; MY: analysis and interpretation of data, drafting the article, final approval; MM: analysis and interpretation of data, drafting the article, final approval; GL: analysis and interpretation of data, drafting the article, final approval; XZ: analysis and interpretation of data, drafting the article, final approval; CYe: study design, analysis and interpretation of data, drafting the article, revising the article critically for important intellectual content, final approval, agreement to be accountable for all aspects of the work; Cyu: study design, drafting the article, revising the article critically for important intellectual content, final approval. All authors contributed to the article and approved the submitted version.
