# Supplementary material for: The expression characteristics of transmembrane protein genes in pancreatic ductal adenocarcinoma through comprehensive analysis of bulk and single-cell RNA sequence
Source: Front Oncol. 2023 May 17;13:1047377. doi: 10.3389/fonc.2023.1047377 (PMC10229874; doi:10.3389/fonc.2023.1047377)
Supplement: Supplementary file 1 [file DataSheet_1.pdf]

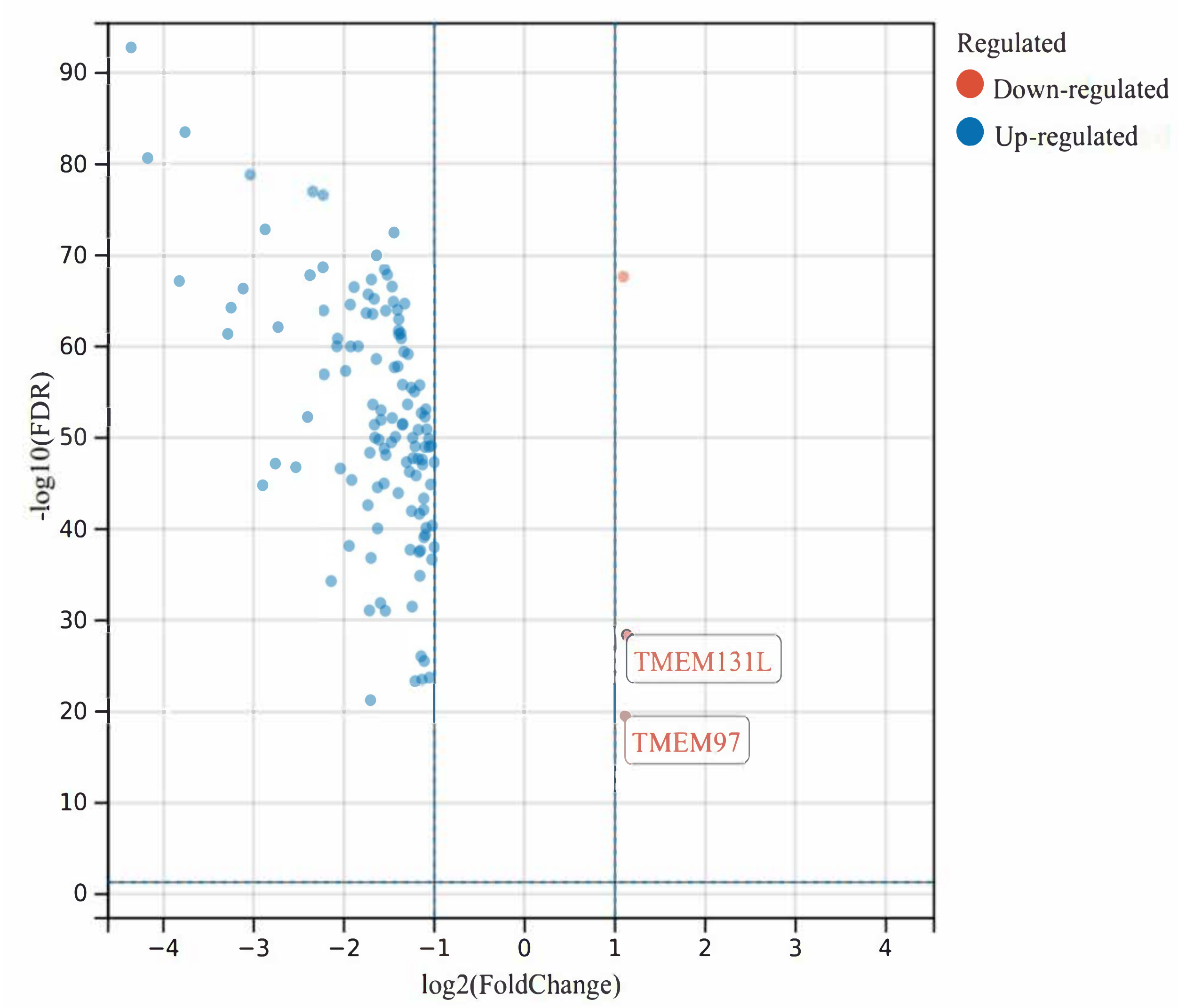

Fig.1S Volcano plot depicted DEGs of TMEM gene through bulk RNA-sequence between normal and tumor tissue. (Up-regulated genes present high expression in tumor tissue and low expression in normal tissues; Down-regulated genes present low expression in tumor tissues and high expression in normal tissues.)

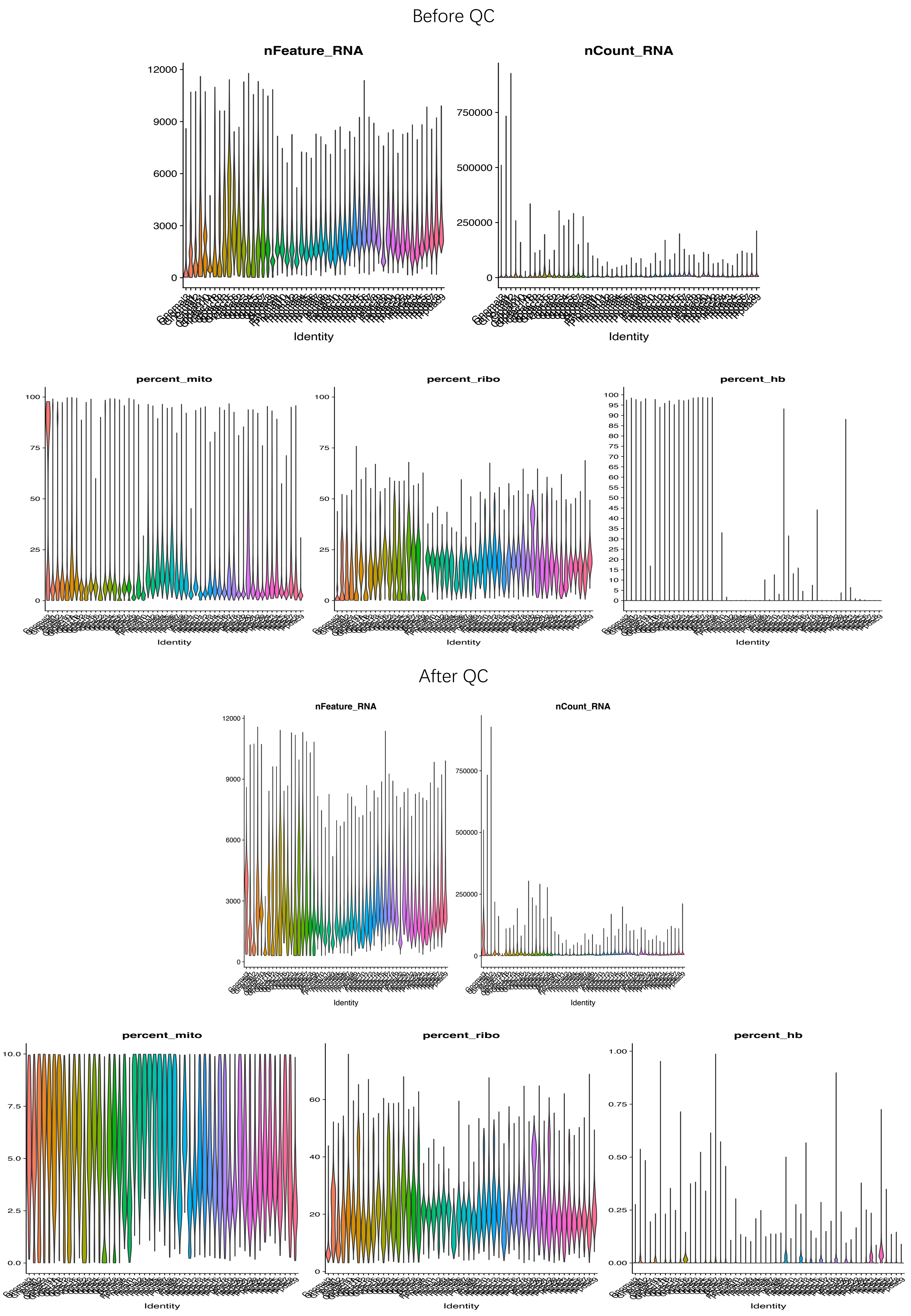

Fig.2S Quality control of single-cell datasets

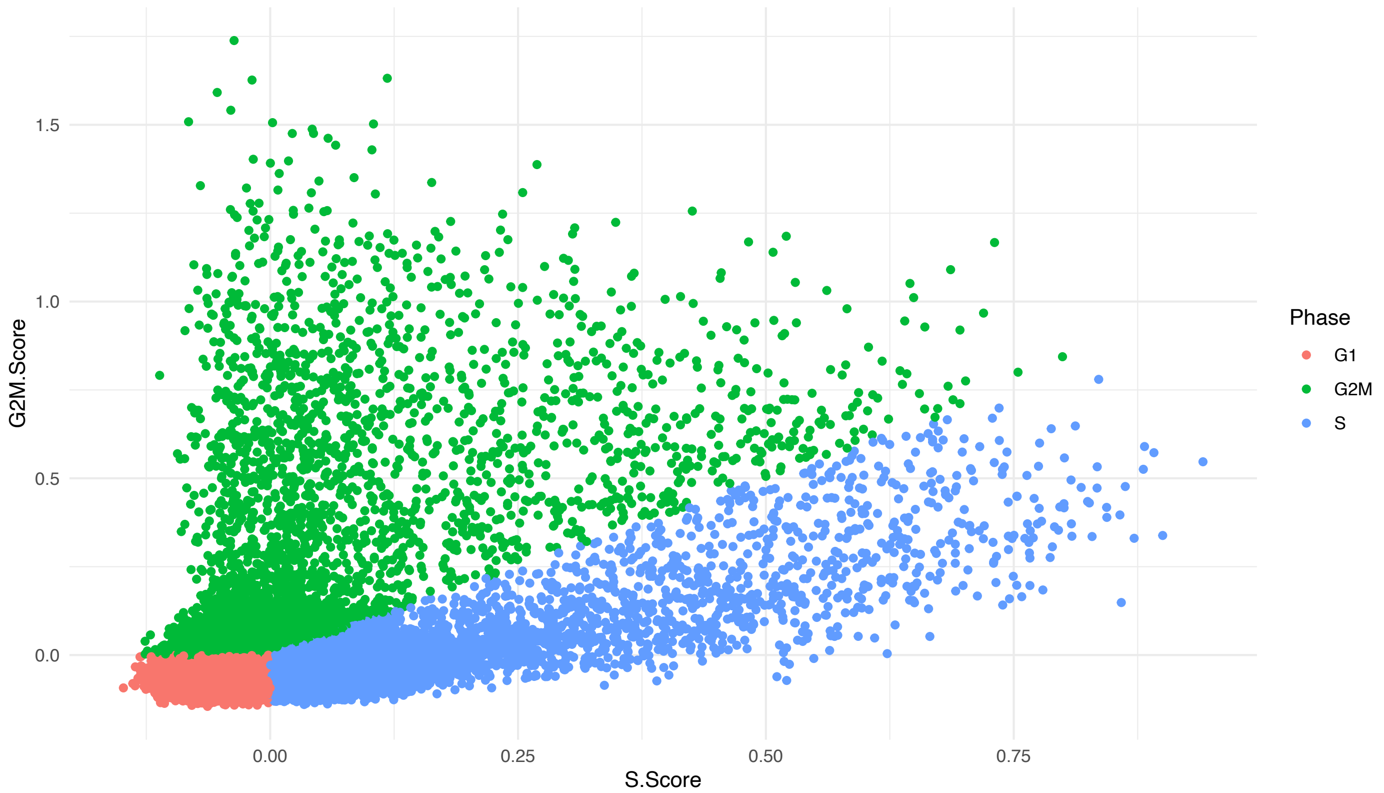

Fig.3S Scatterplot depicted the cells in different the cell cycle phase by single-cell analysis.

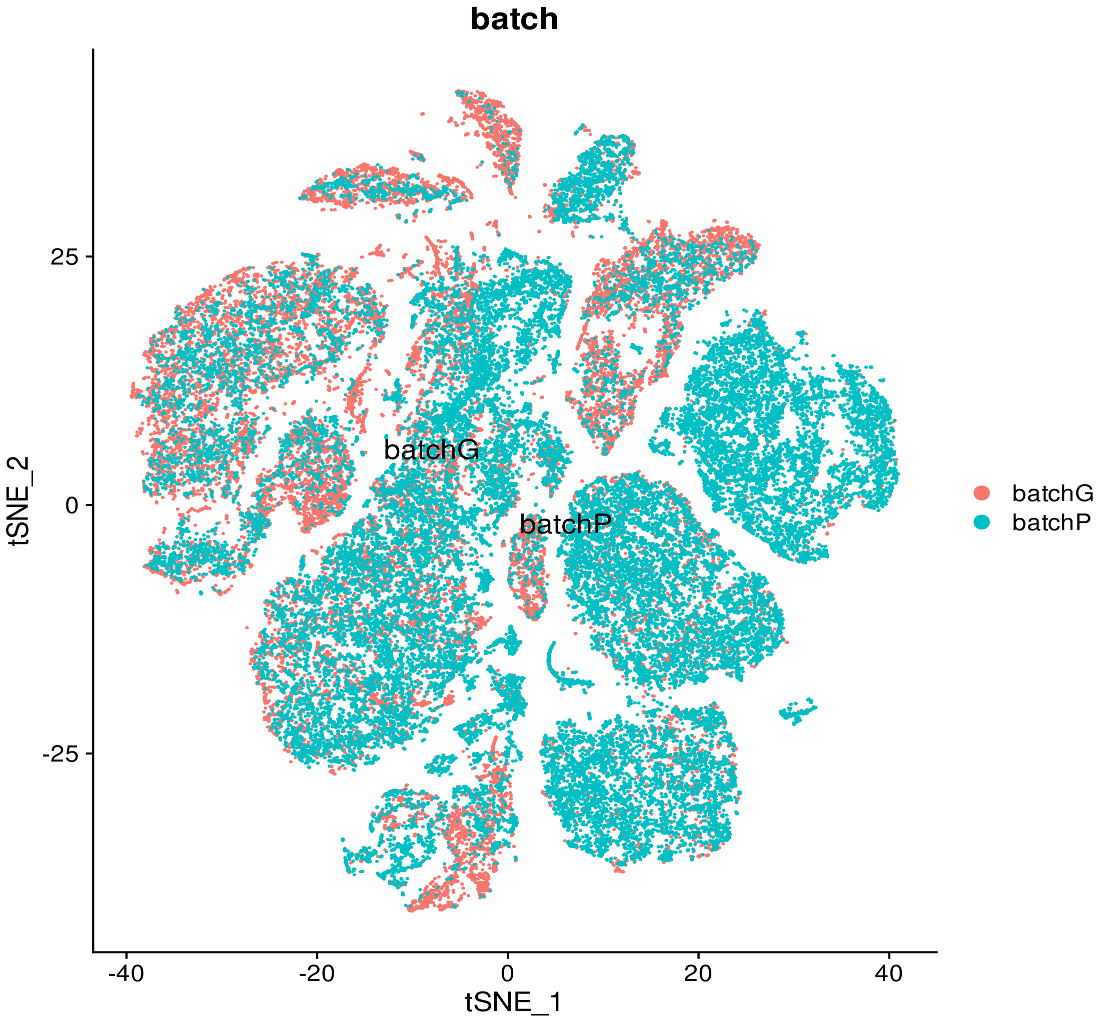

Fig.4S Batch effect evaluation by tSNE reduction plot after datasets integration

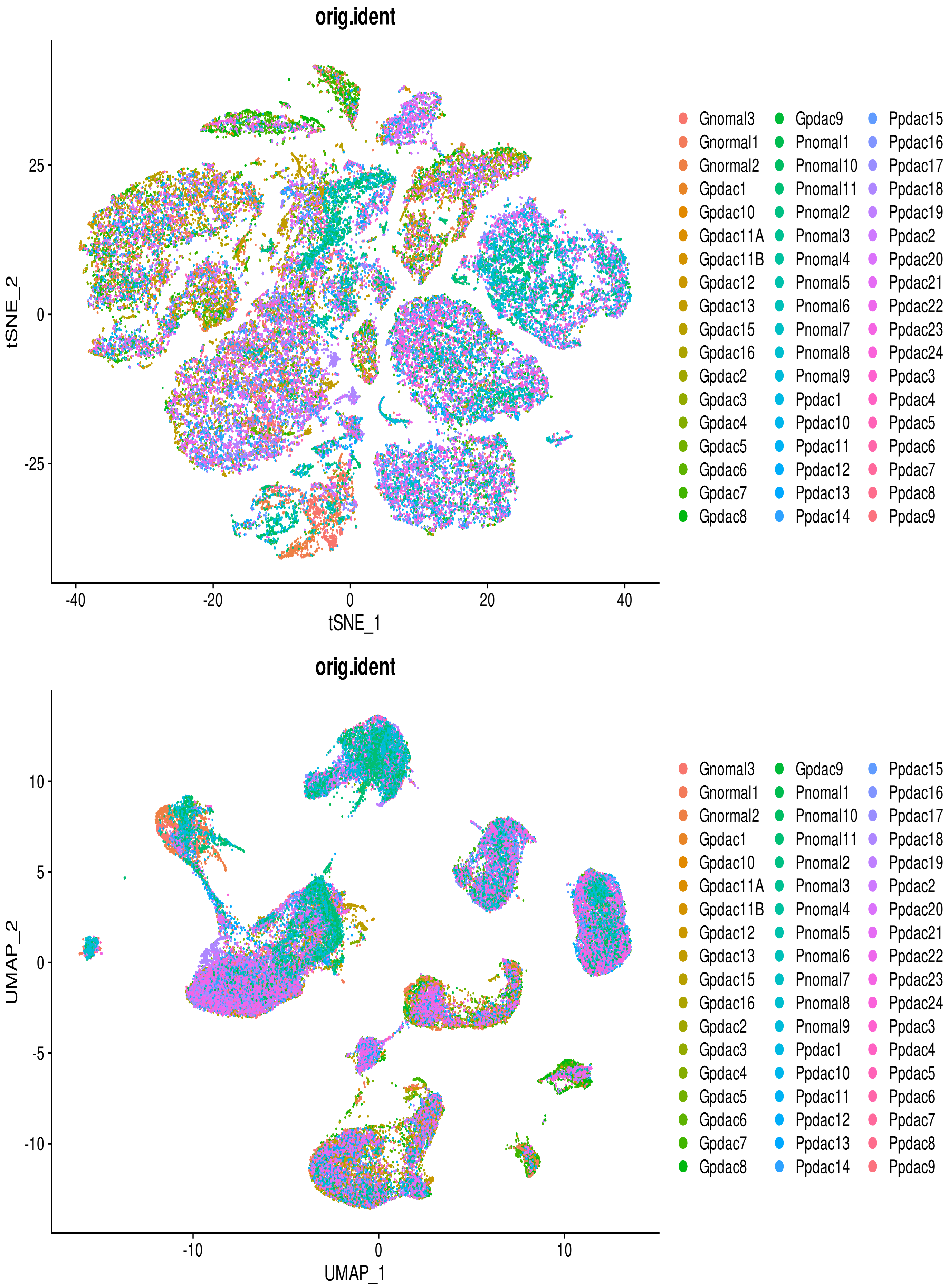

Fig.5S tSNE and UMAP reduction plot of cells labeled by each included samples

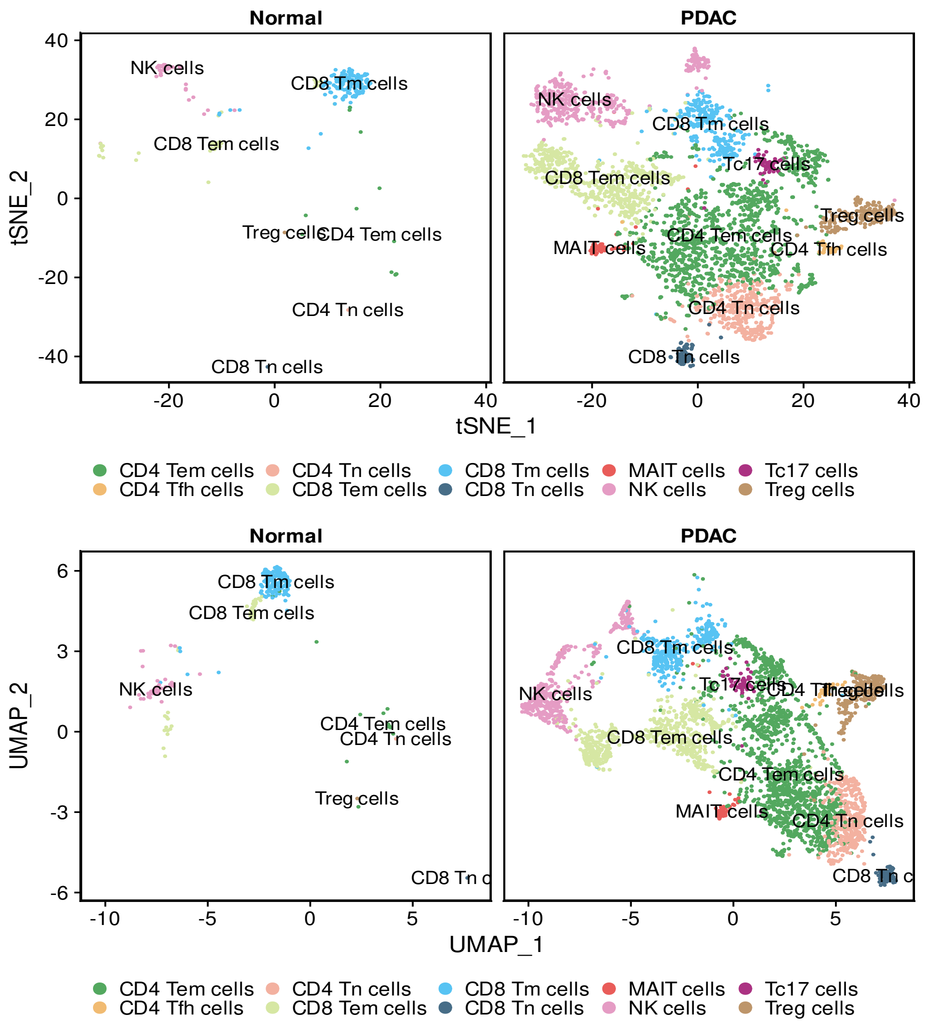

Fig.6S UMAP and tSNE plot revealed subgroups of T cells.

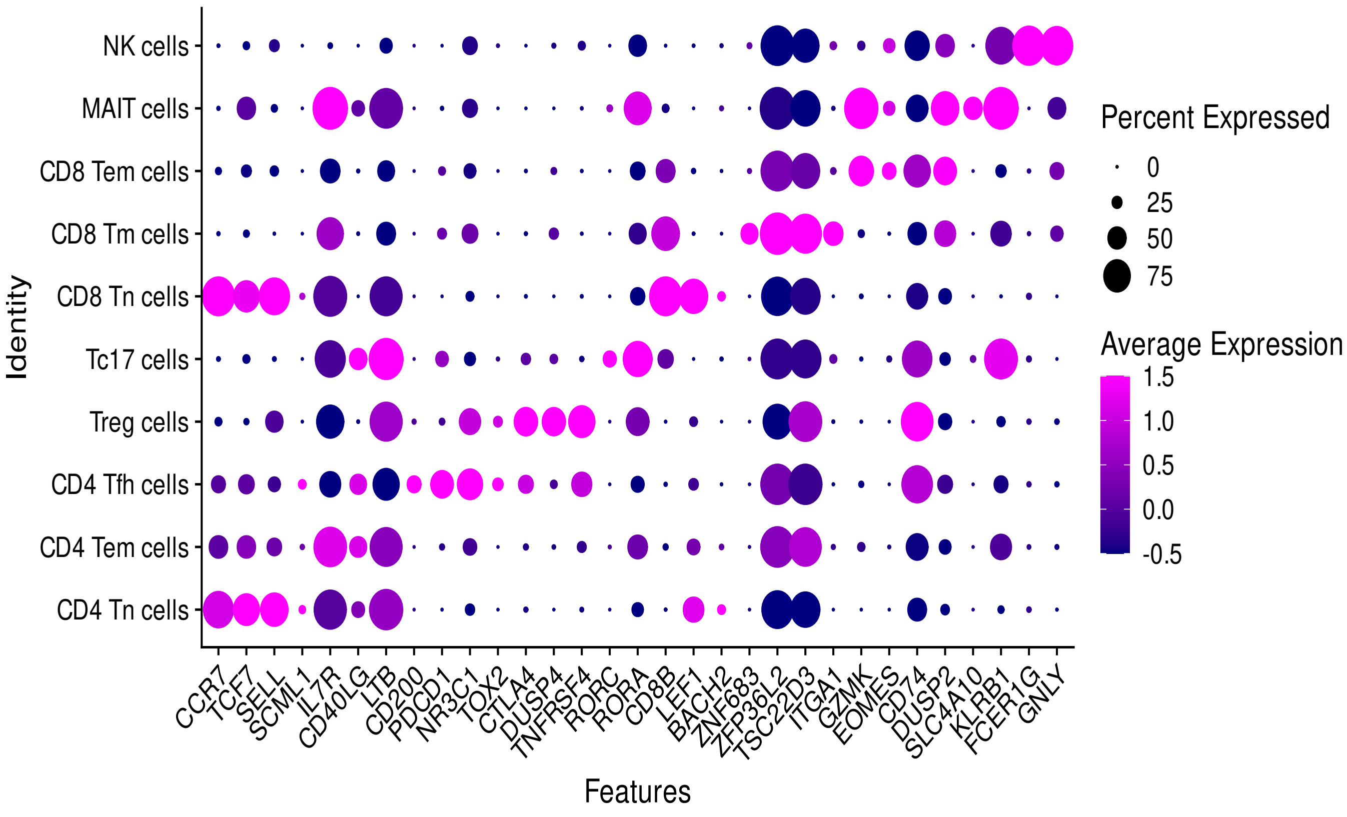

Fig.7S Marker gene of T cell subgroups.

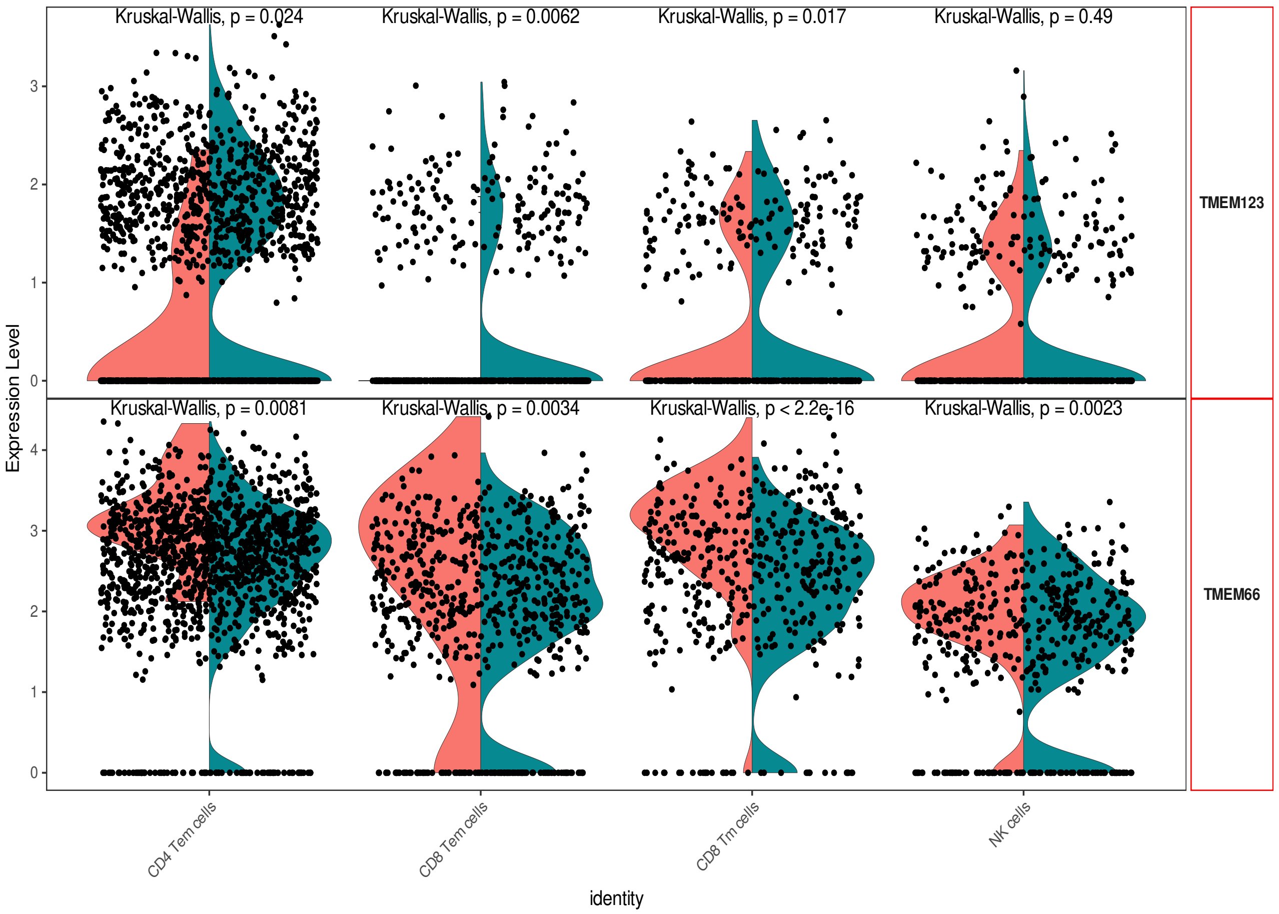

Fig.8S Difference of TMEM gene expression in T cells

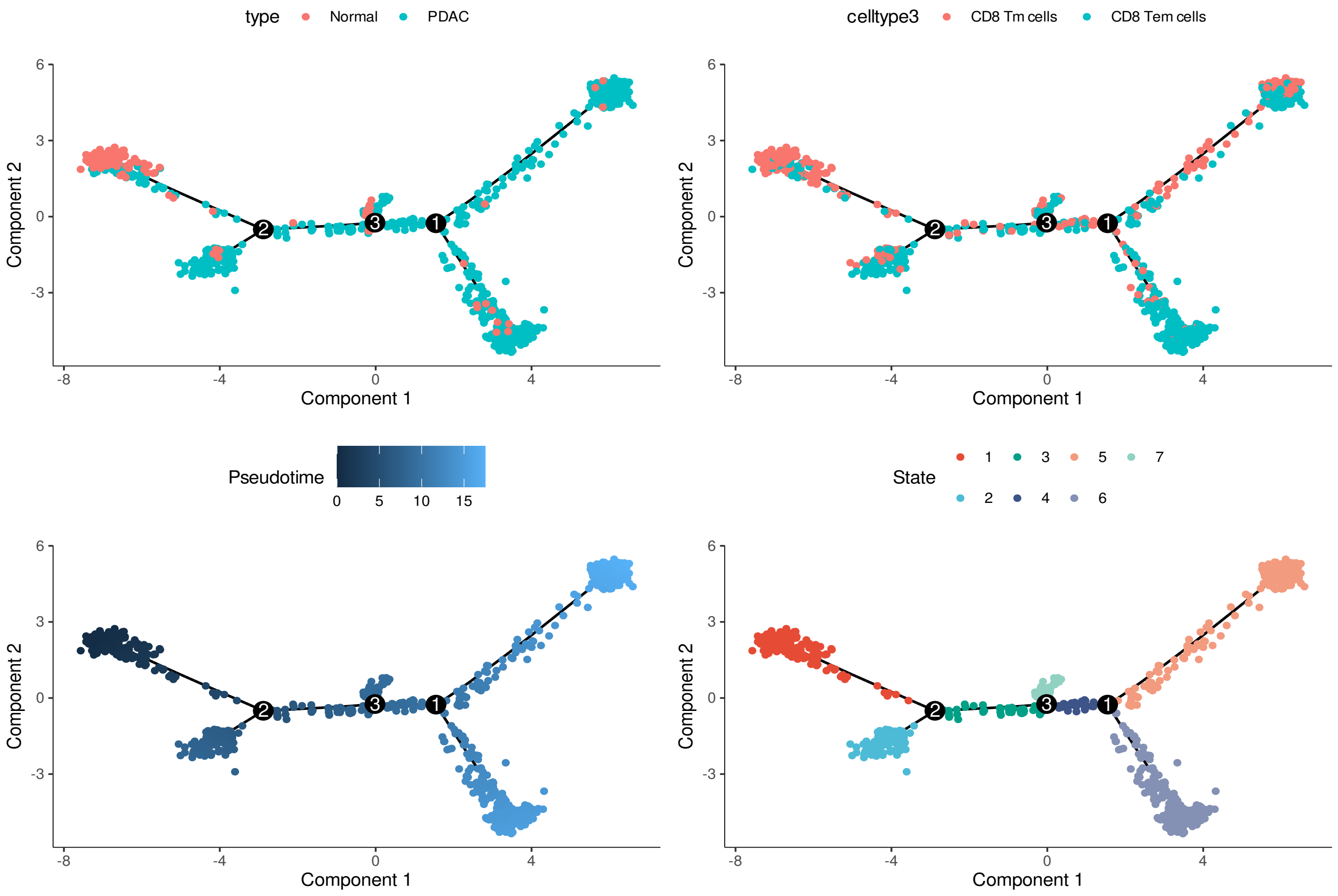

Fig.9S Pseudotime-analysis revealed the trajectory from CD8 T memory cells to CD8 effector memory cells.

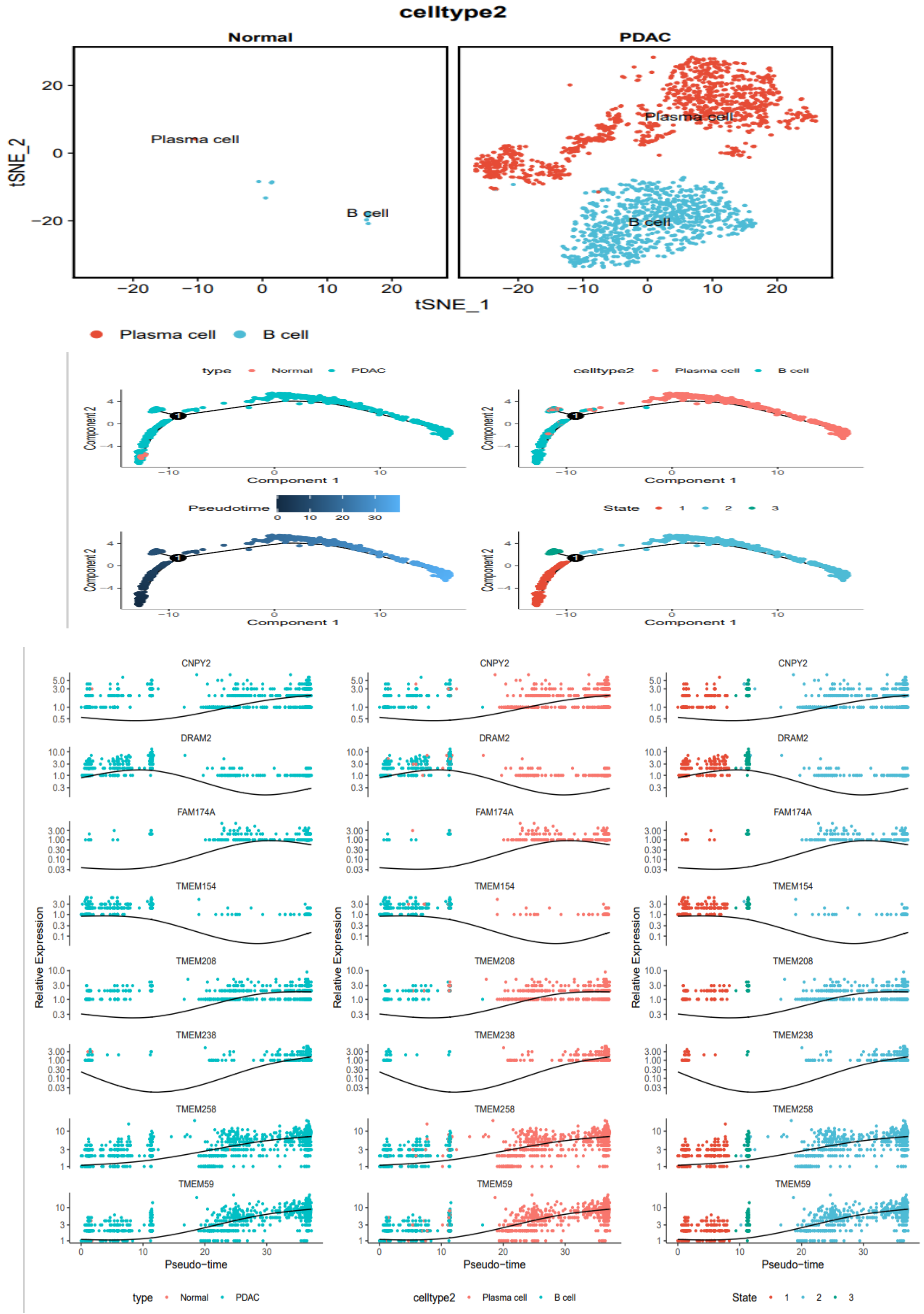

Fig.10S B cell lineage analysis revealed the distribution of subgroups, pseudo-time trajectory and TMEM gene expression through pseudo-time.

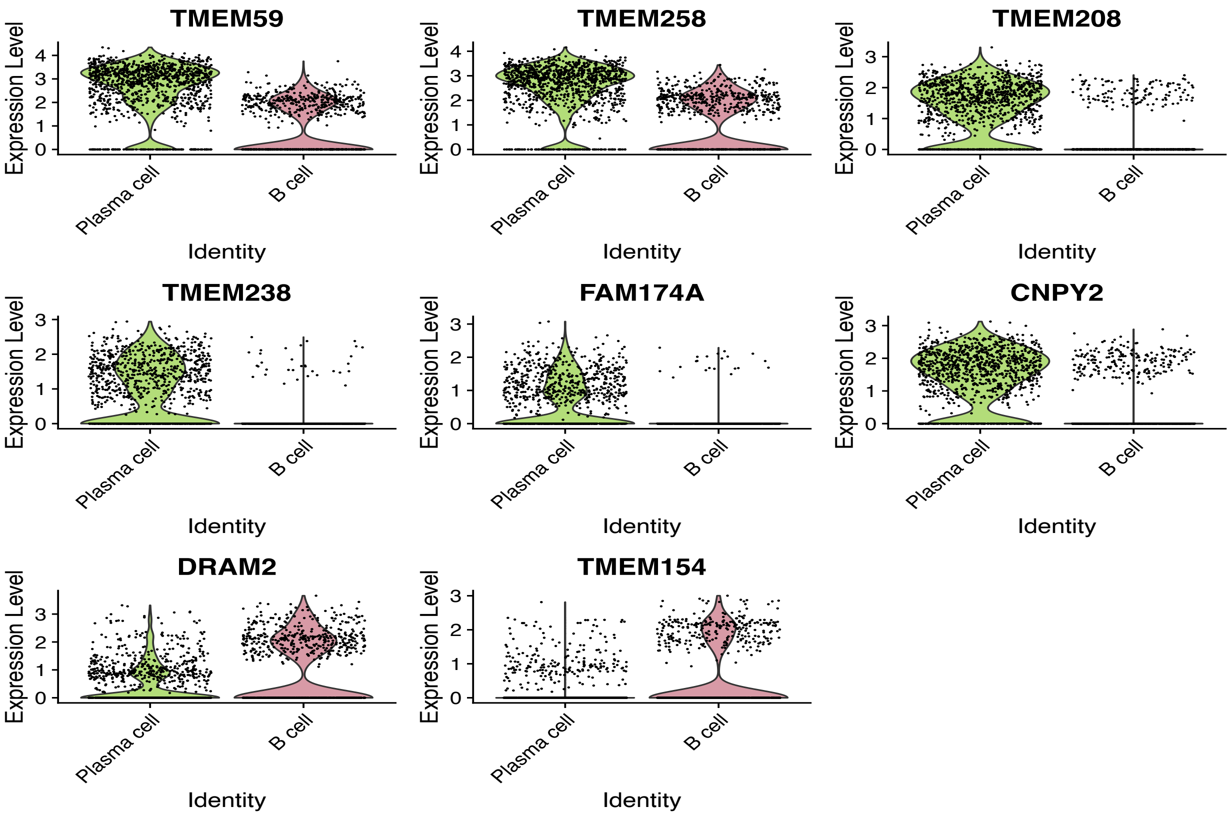

Fig.11S Difference of TMEM gene expression in B cell lineage.

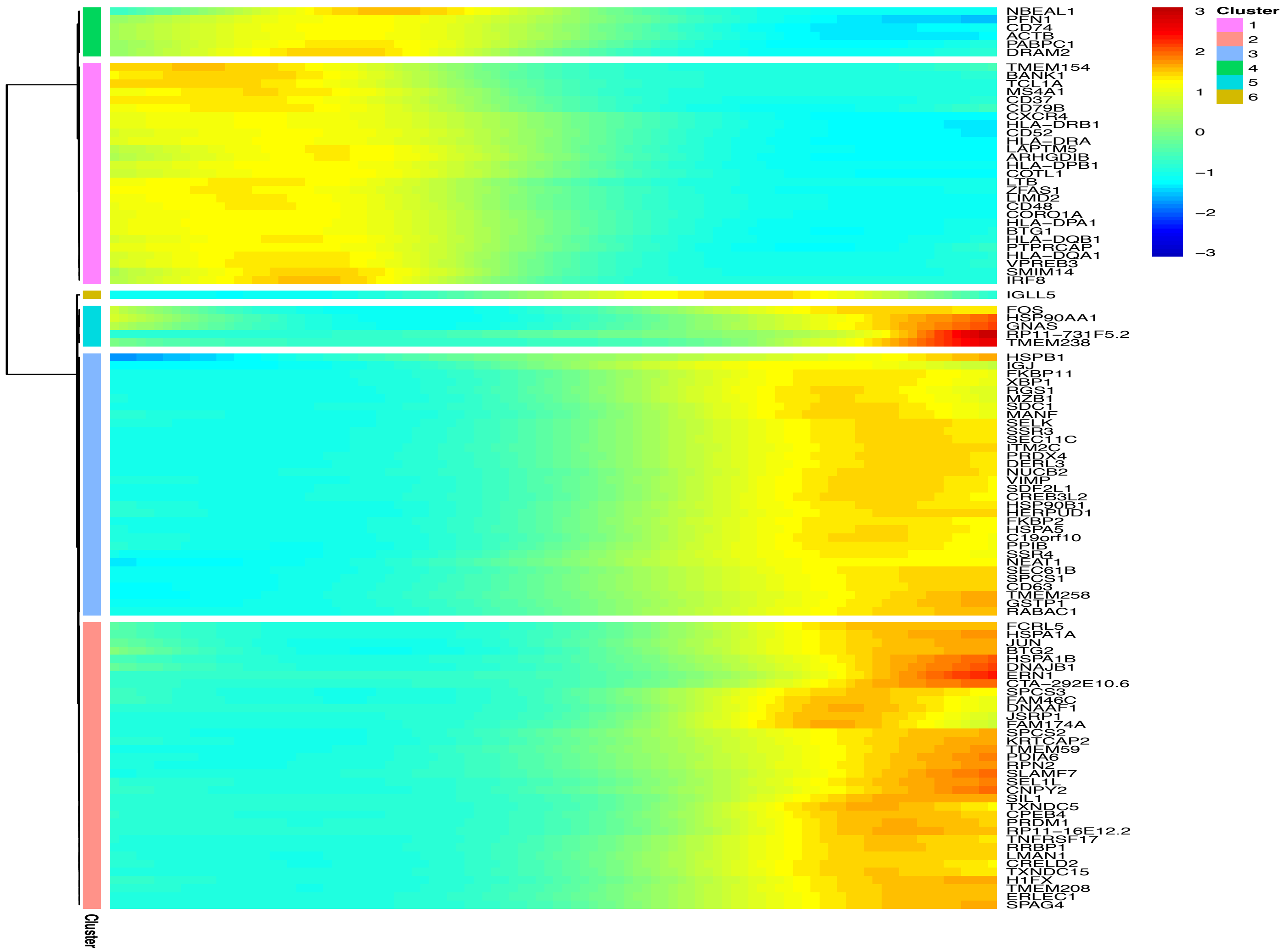

Fig.12S Heatmap revealed the characteristics of TMEM gene expression follow the pseudo-time.

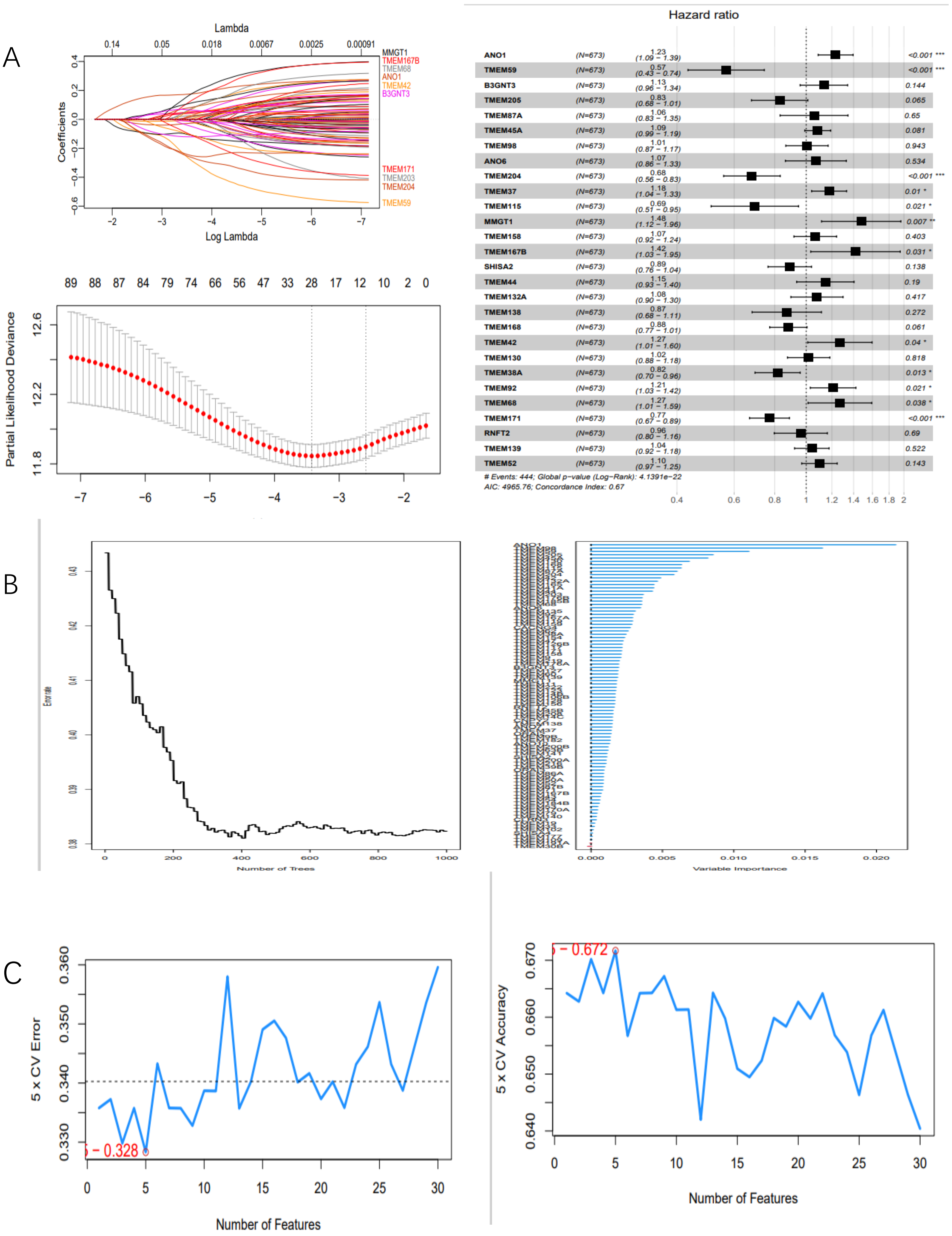

Fig.13S Lasso regression analysis (A), Random Forest survival analysis (B), and Support Vector Machine Recursive Feature Elimination analysis (C) to select the prognostic TMEM gene.

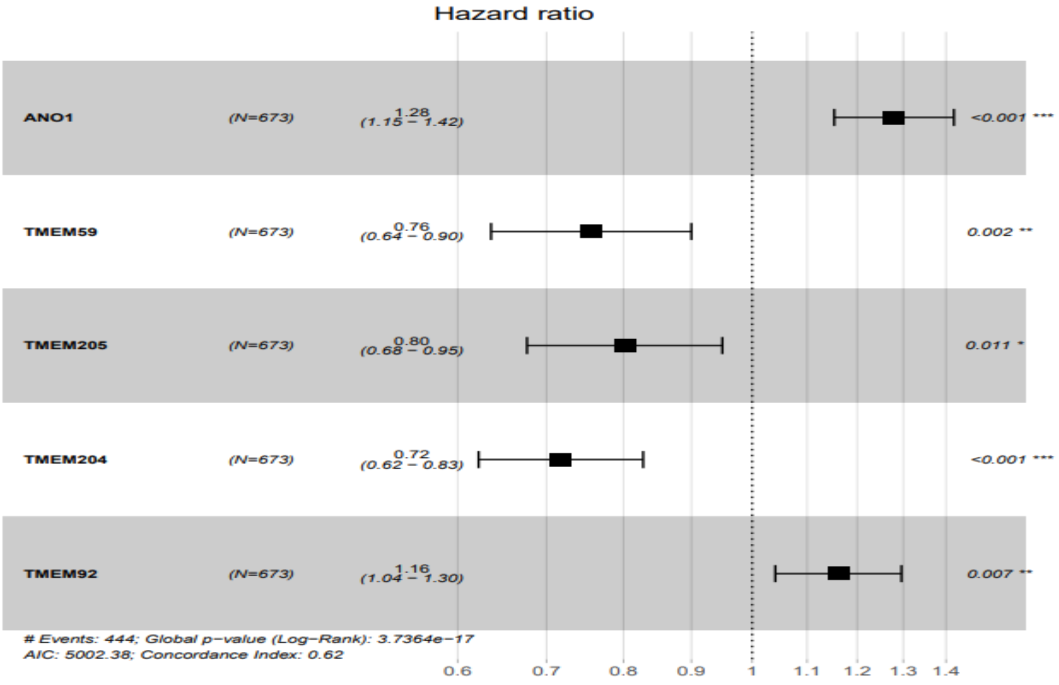

Fig.14S Cox-regression analysis of 5 key TMEM gene based on internal analytic cohort.

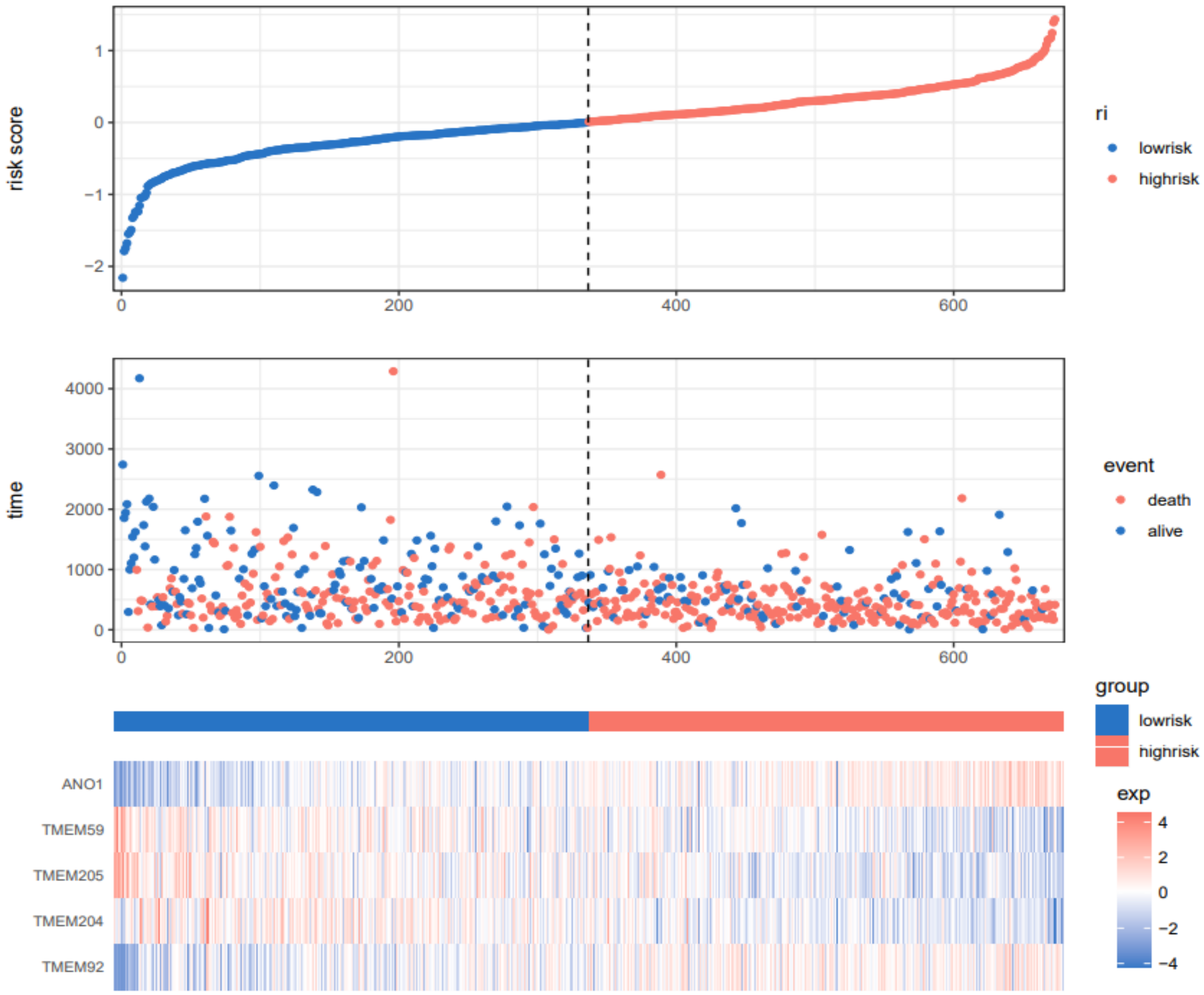

Fig.15S Combined plot of TMEM risk-score based on the outcomes of internal bulk RNA-seq datasets.

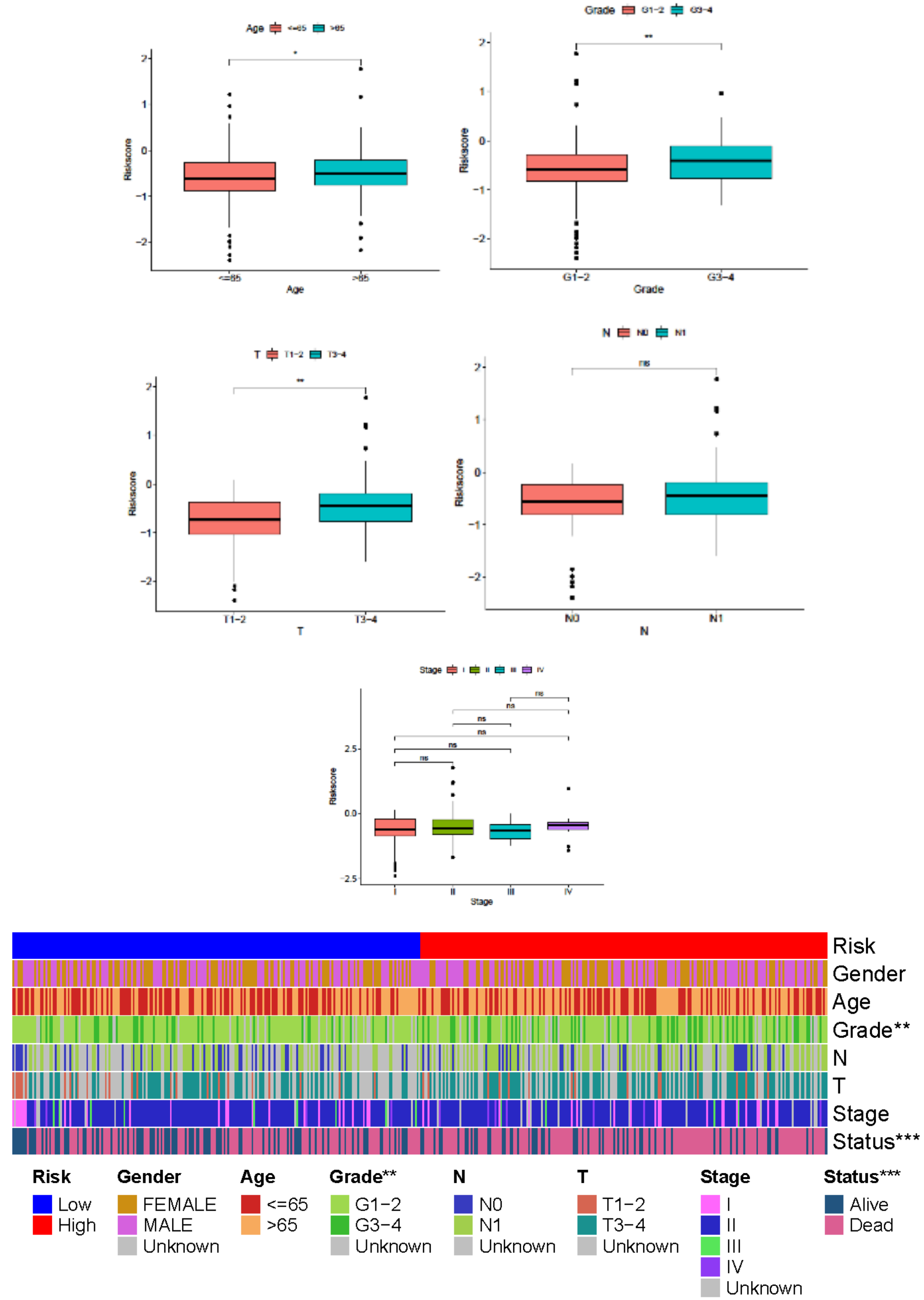

Fig.16S Combined plot revealed correlation between clinical case indicators and TMEM risk score in TCGA cohort

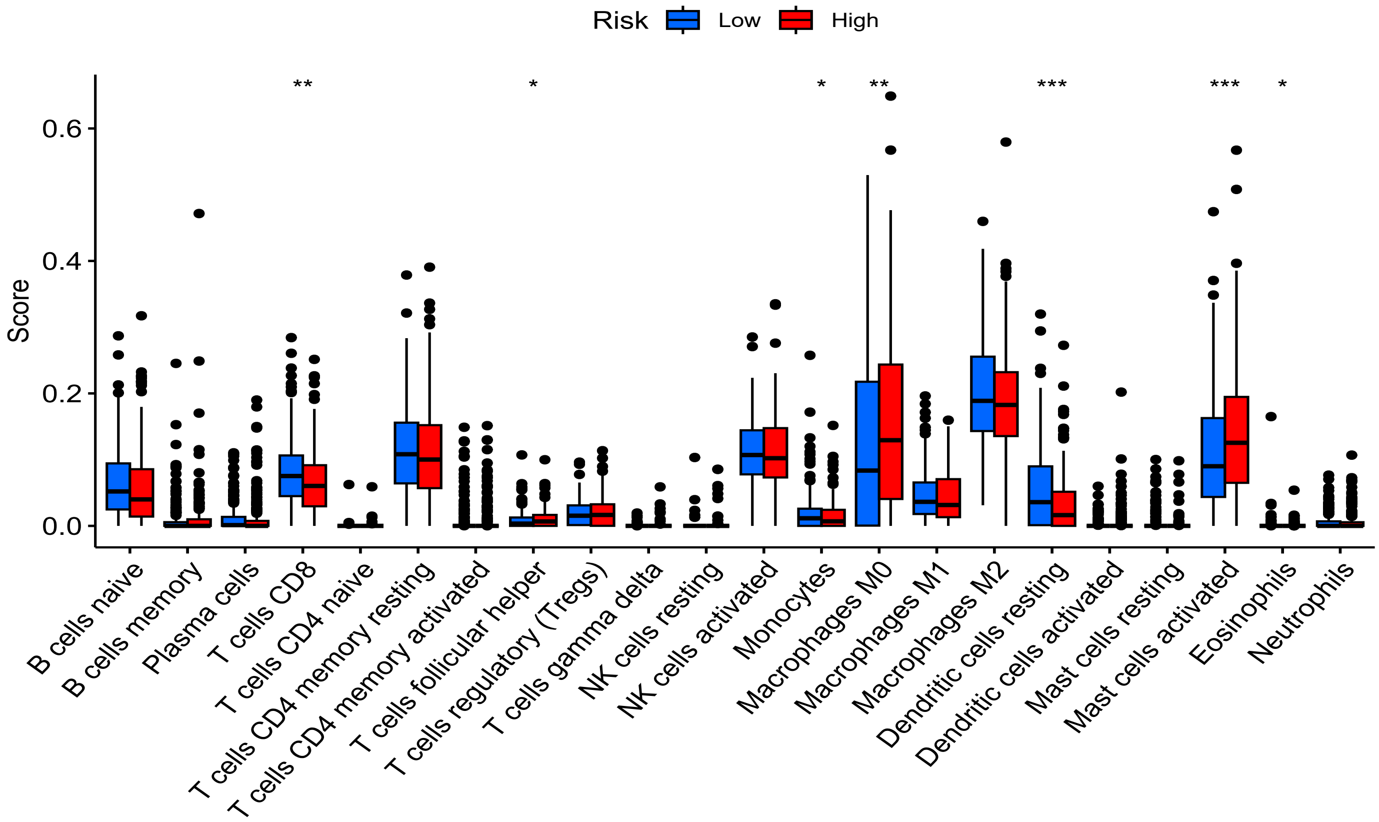

Fig.17S CIBERSORT algorithm revealed distinct immune cell infiltration between two TMEM riskscore groups.

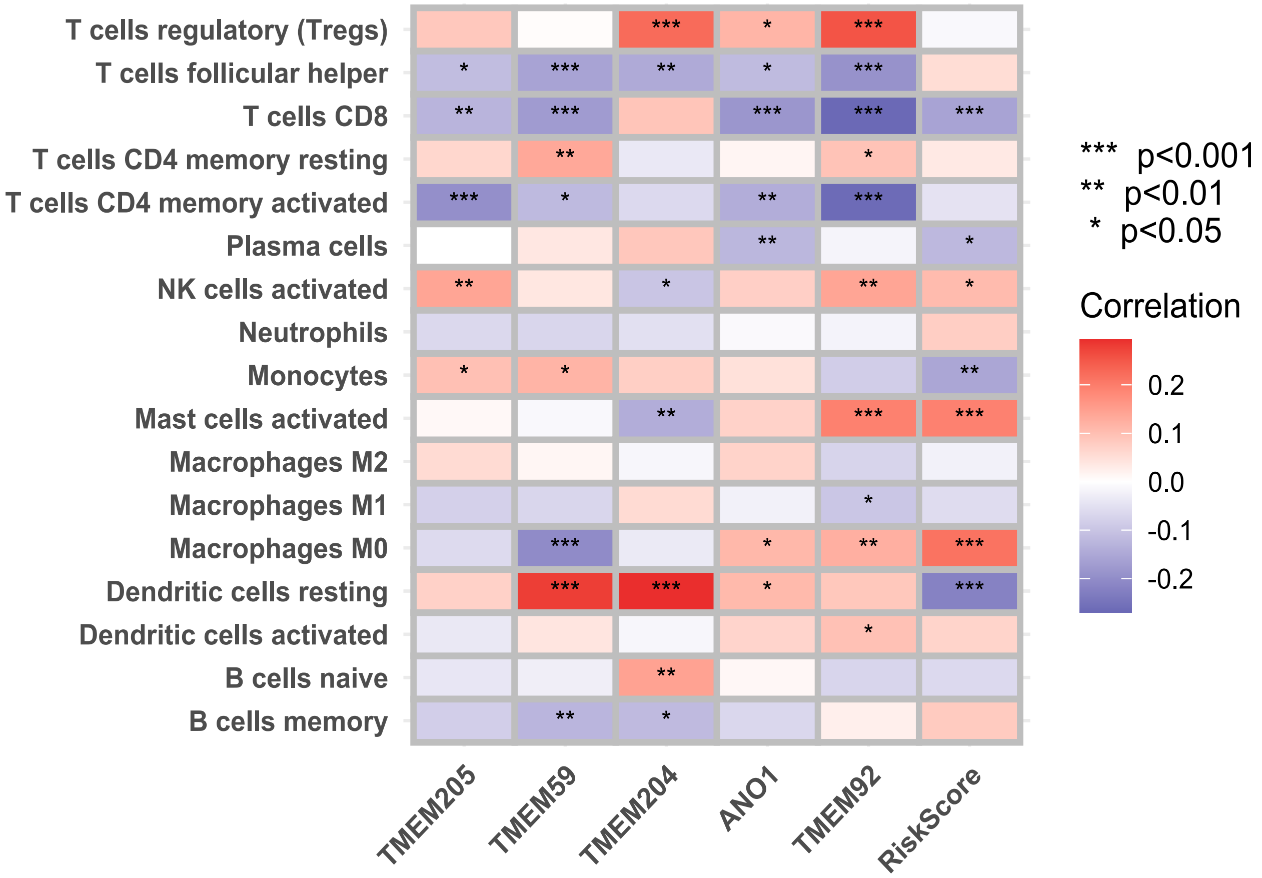

Fig.18S Heatmap depicted the correlation between 5 key TMEM genes and immune cell infiltration in PDAC.

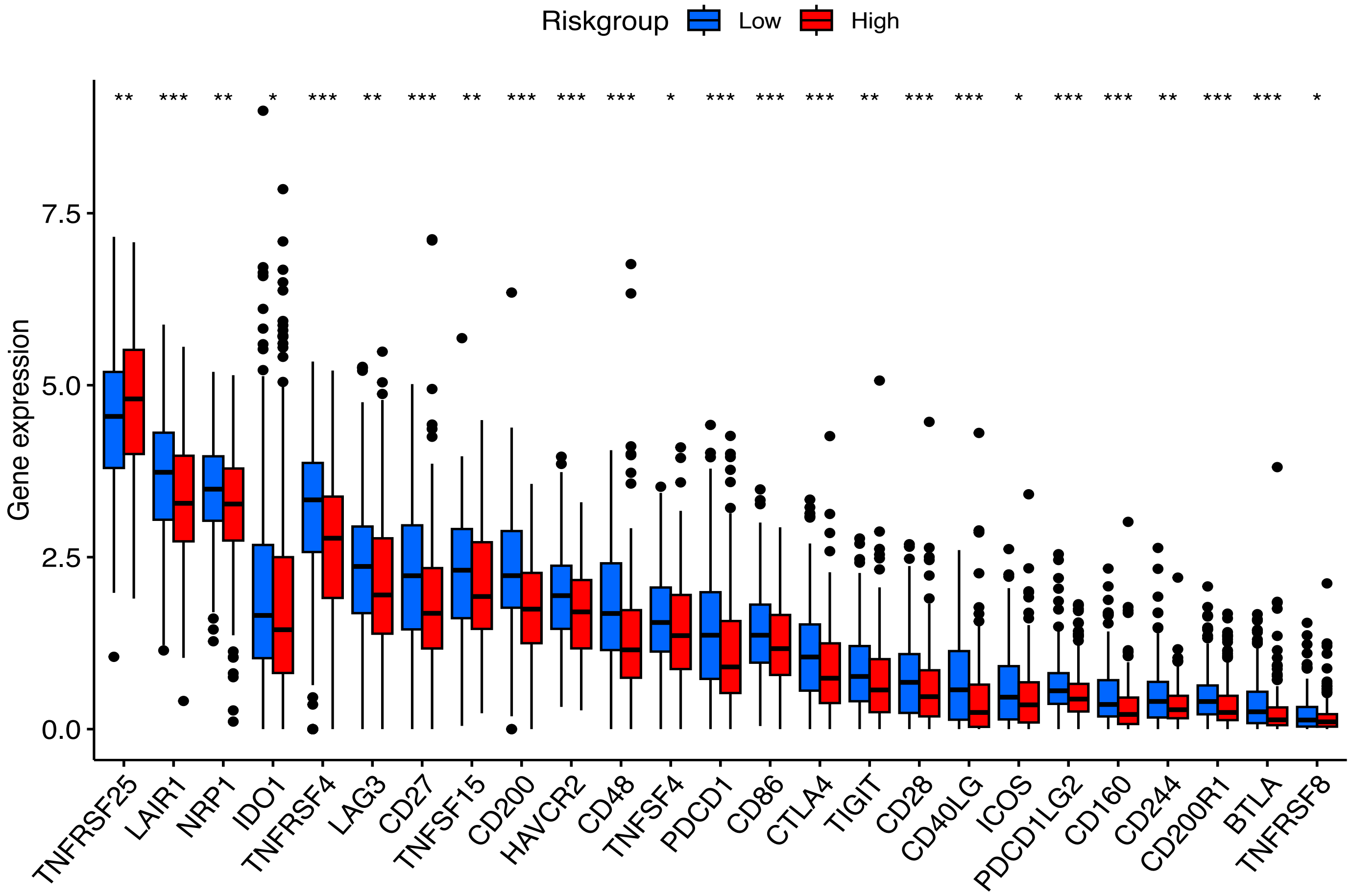

Fig.19S Significant disparities in various immune checkpoint genes between two TMEM riskscore groups.

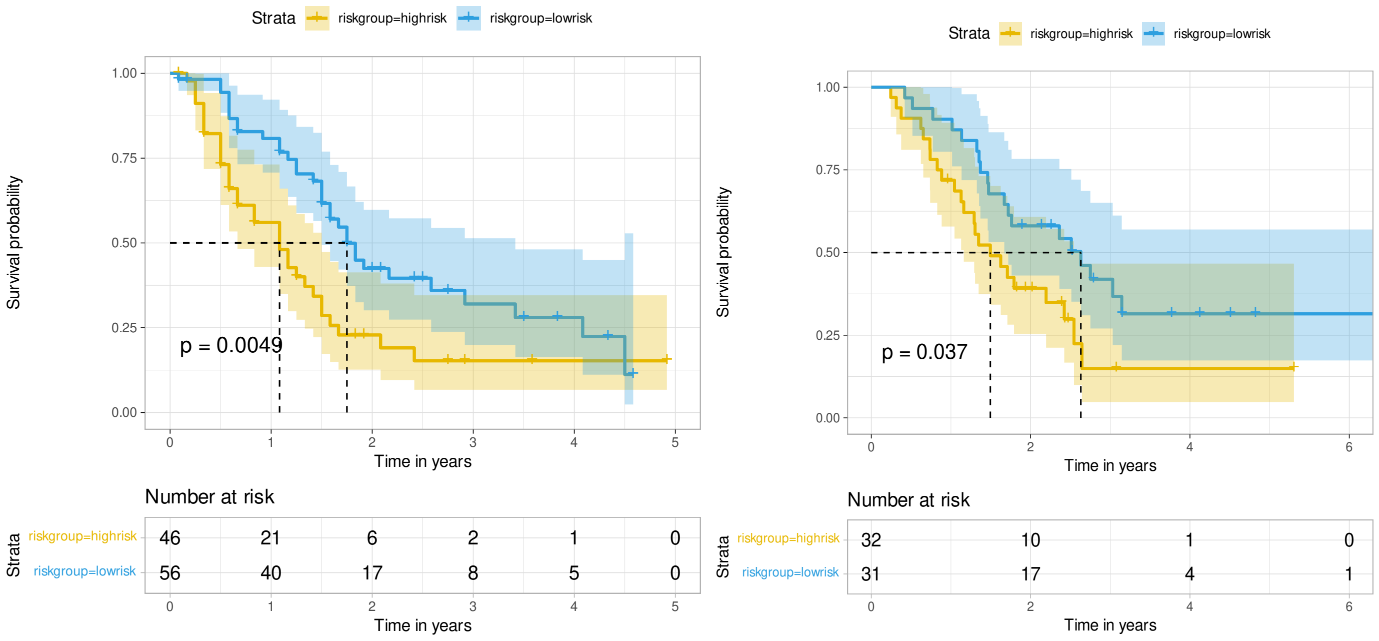

Fig.20S KM plot of OS in GSE21501(left) and GSE57495(right) patients

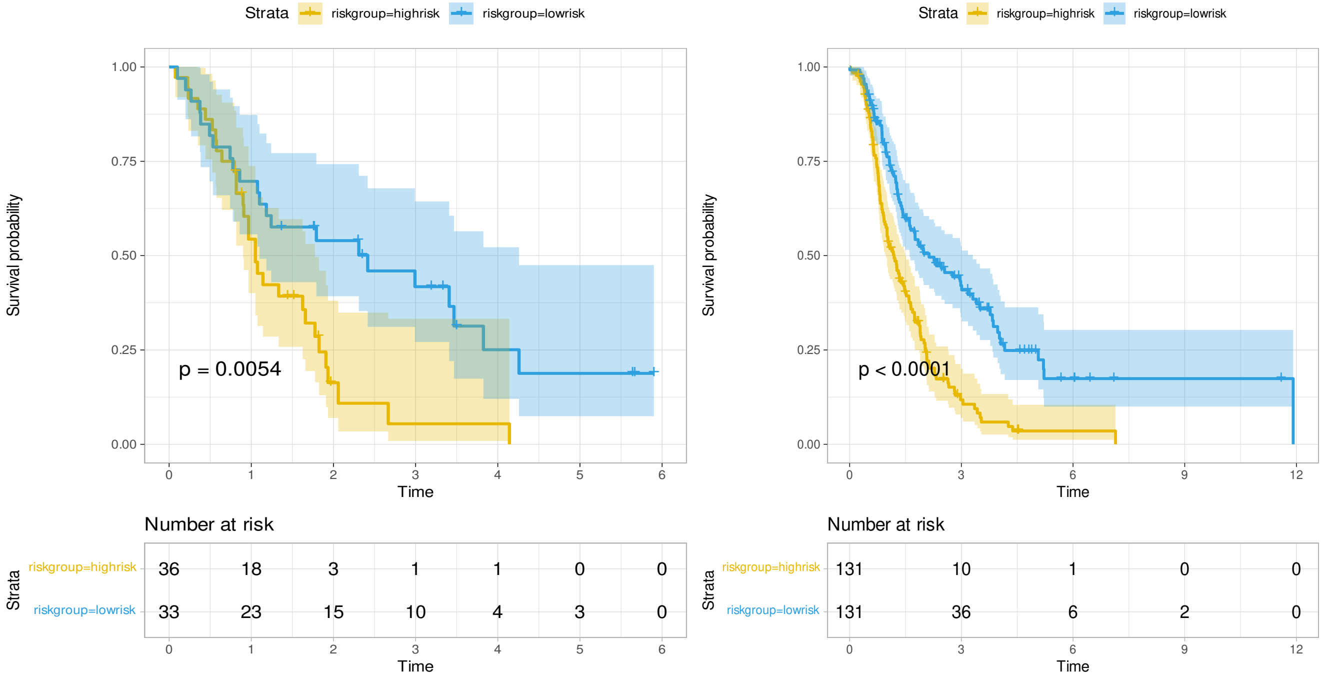

Fig.21S KM plot of OS in GSE62452(left) and ICGC-PAAD (right) patients

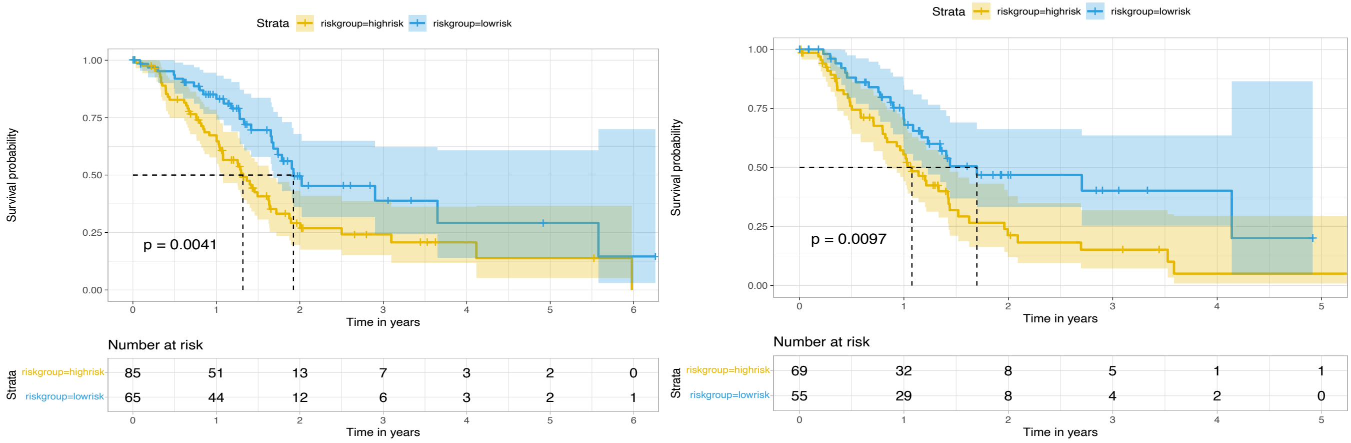

Fig.22S KM plot of OS (left) and DFS (right) in TCGA-PAAD patients

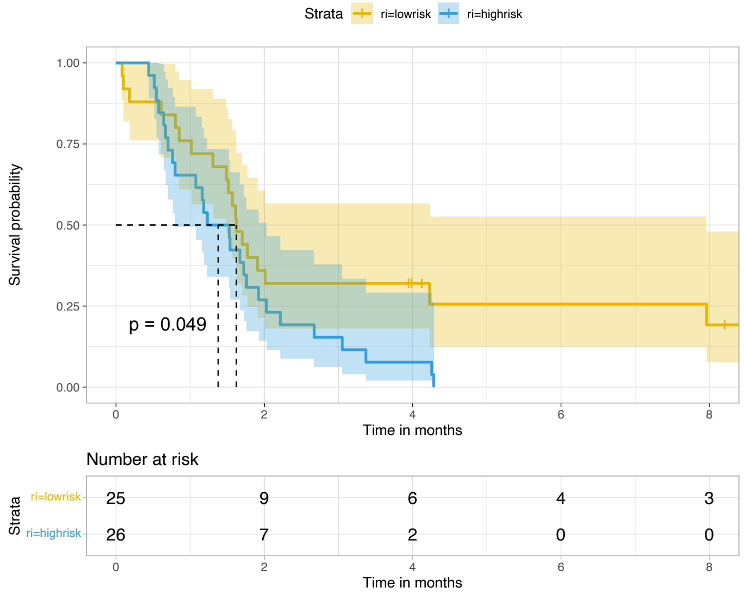

Fig.23S KM plot of OS in GSE79668 patients

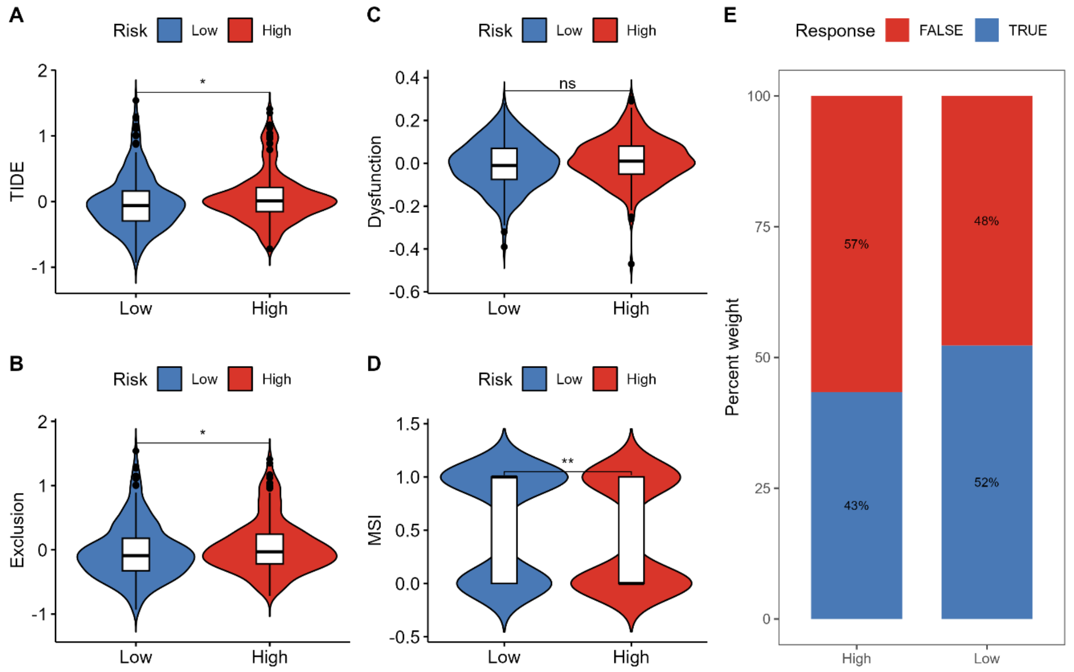

Fig.24S TIDE algorithm predicted the therapeutic response of ICIs treatment. (A-D) TIDE score, immune dysfunction score, immune exclusion score and Microsatellite Instability score between two TMEM riskscore group. (E) Bar-plot showed the predicted response to ICIs treatment through TIDE algorithm.

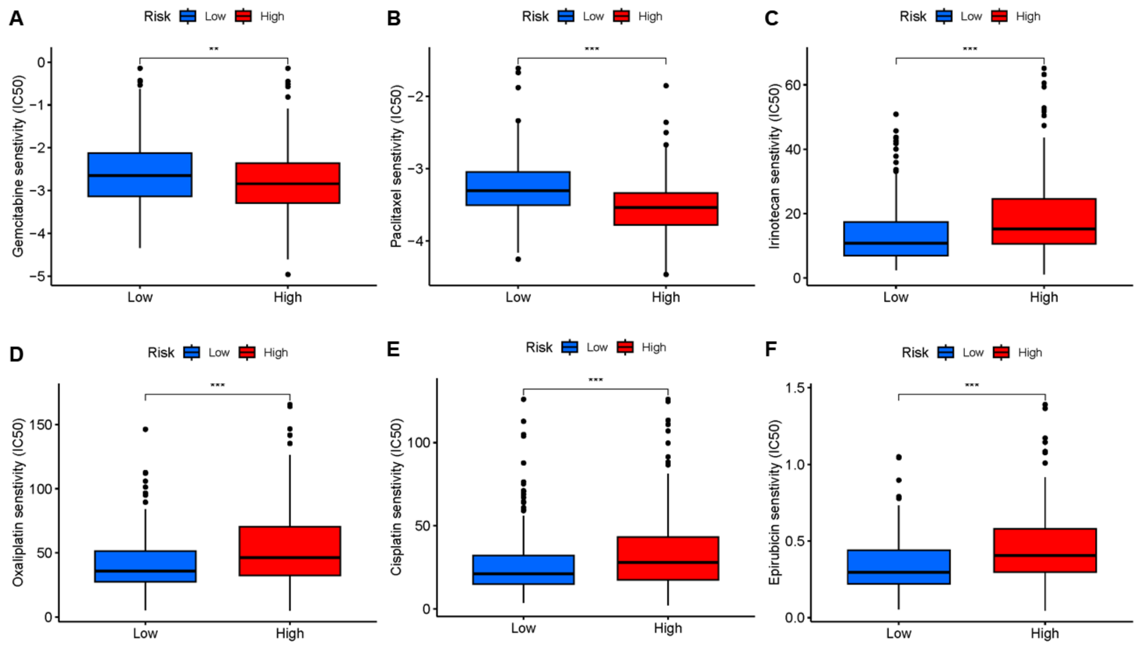

Fig.25S The prediction of IC50 value for common drugs used in PDAC to compare their treatment sensitivities, including: Gemcitabine, Paclitaxel, Oxaliplatin and Cisplatin. However, no significance observed in 5-Fu.

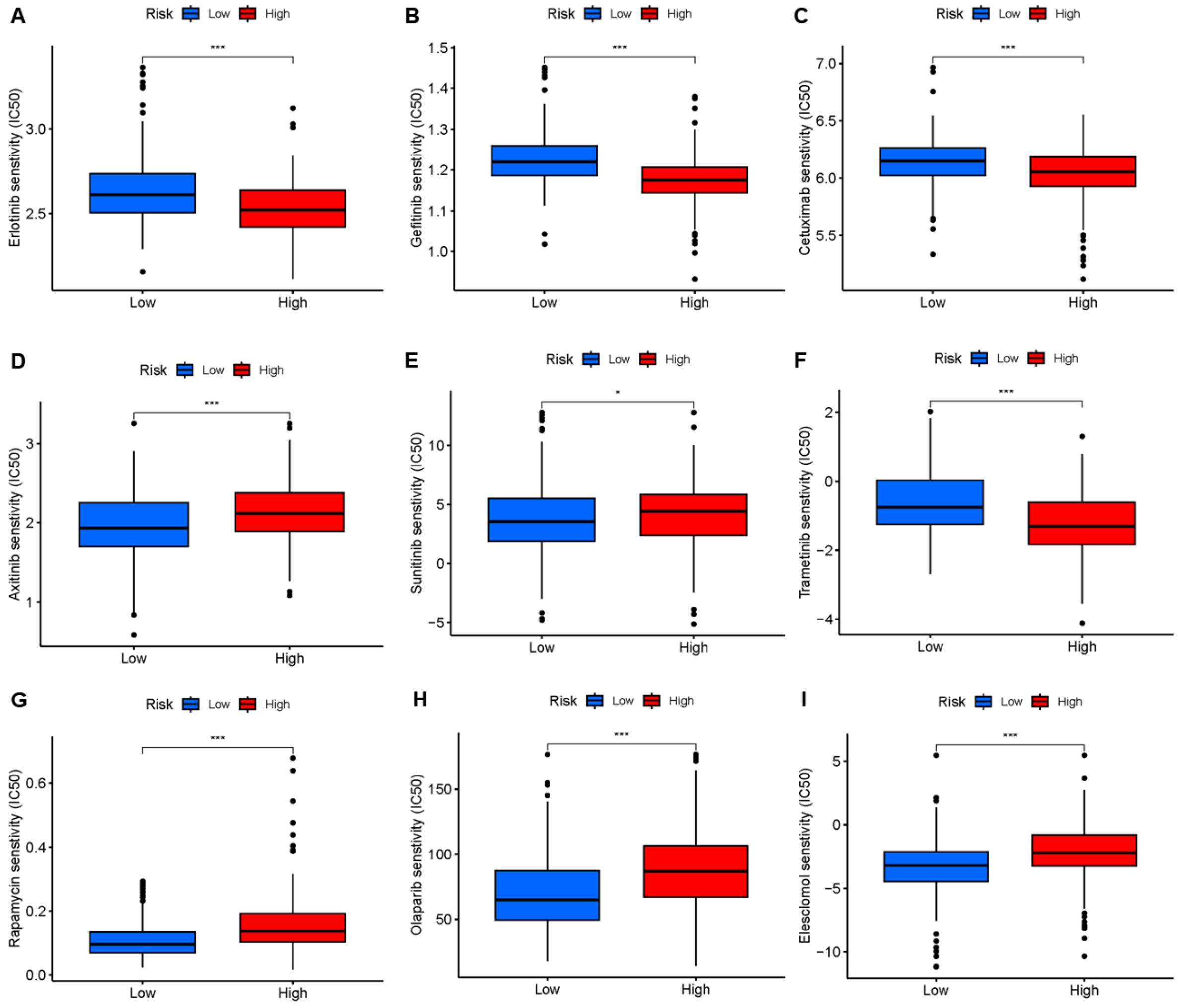

Fig.26S The prediction of IC50 value for drugs might have potential use for PDAC based on TMEM riskscore groups.

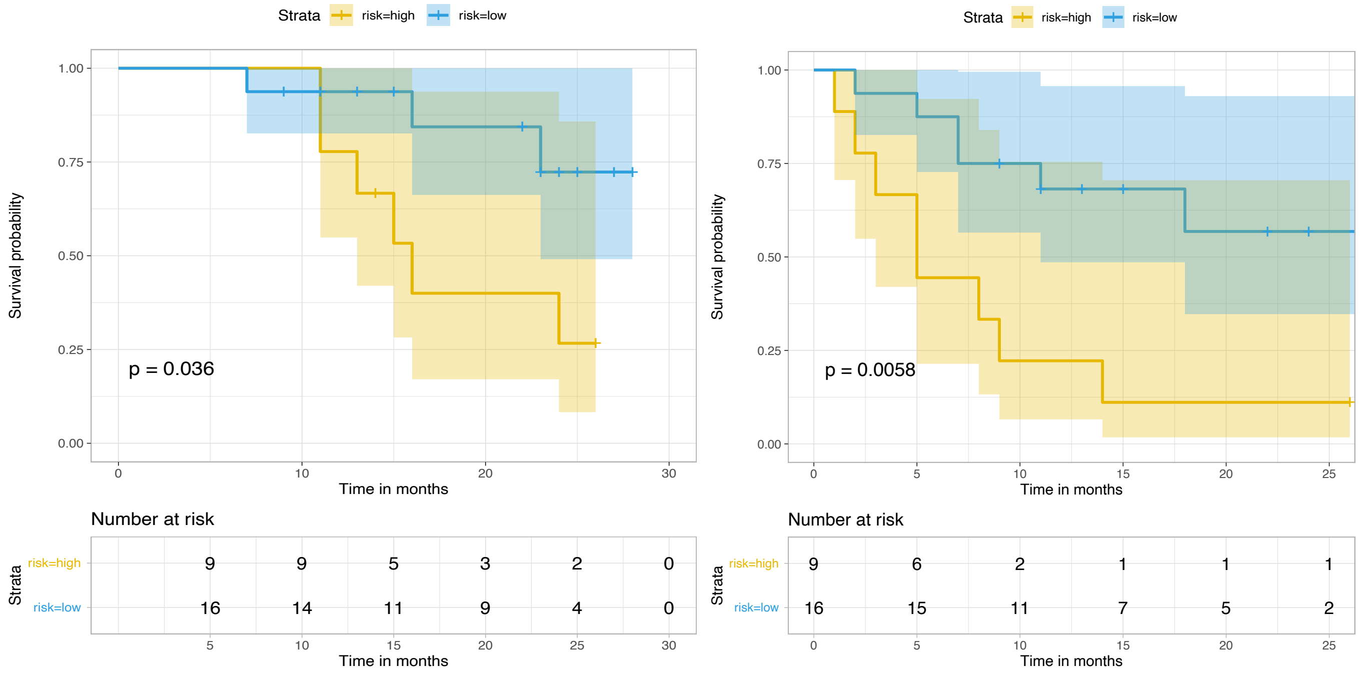

Fig.27S KM plot of OS (left) and DFS (right) in patients received gemcitabine-based adjuvant chemotherapy

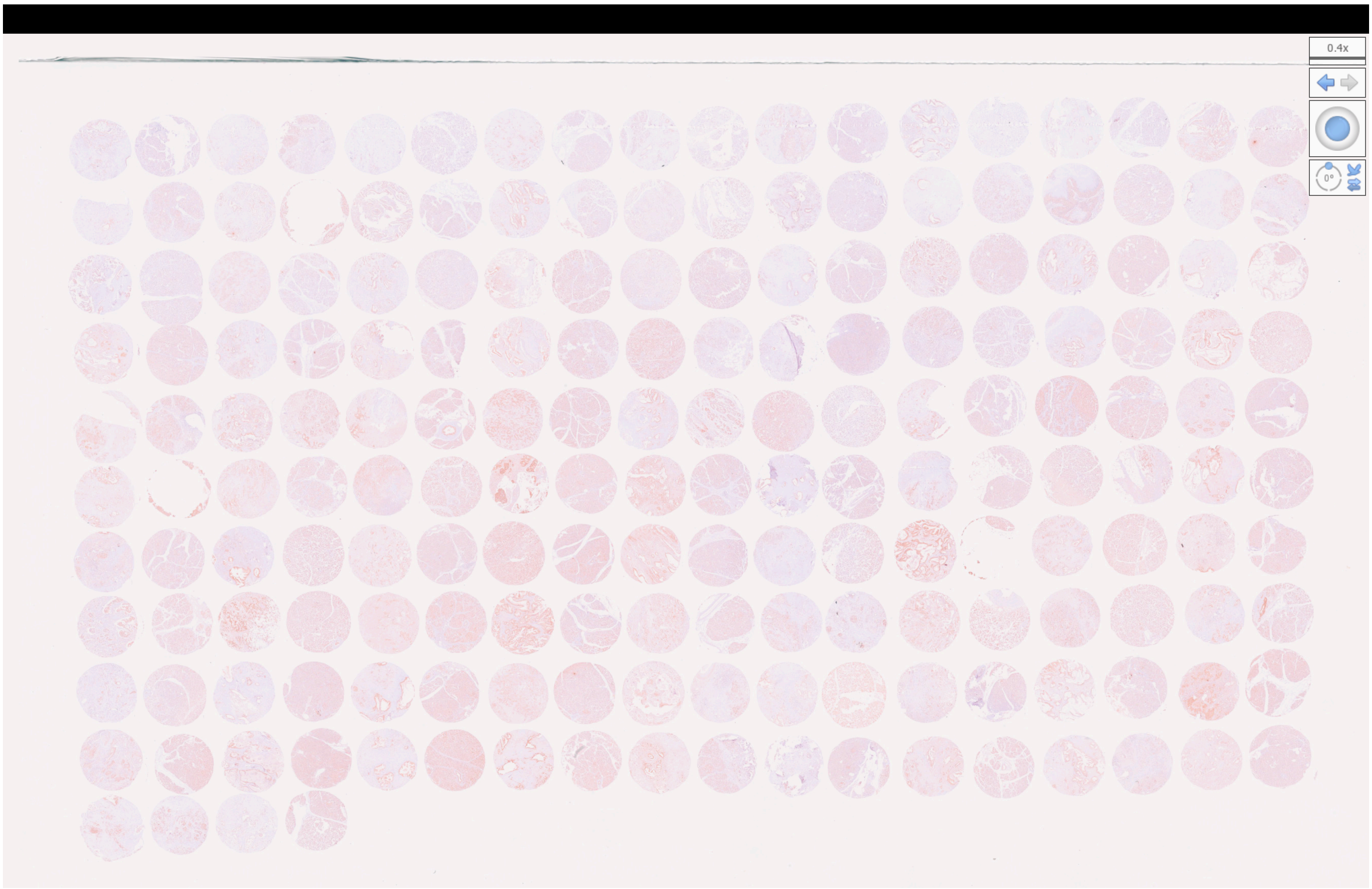

Fig.28S Overview of IHC stanning of TMEM92 on tissue matrix array.

Sample collection

All post-operative PDAC tissues and were collected from the Department of General Surgery, Peking University Third Hospital (Beijing, China), with Institutional Review Board approval (M2016361) and written informed consents from all subjects. The enrolled patients were all diagnosed and these patients had not received chemotherapy or radiotherapy prior to surgery. The fresh tumor tissues from the surgical samples were immediately preserved in liquid nitrogen and then used for further RNA-sequence.

RNA quantification and qualification

RNA integrity was assessed using the RNA Nano 6000 Assay Kit of the Bioanalyzer 2100 system (Agilent Technologies, CA, USA).

Library preparation for Transcriptome sequencing

A total amount of 1 μg RNA per sample was used as input material for the RNA sample preparations. Briefly, mRNA was purified from total RNA using poly-T oligo-attached magnetic beads. Fragmentation was carried out using divalent cations under elevated temperature in First Strand Synthesis Reaction Buffer(5X). First strand cDNA was synthesized using random hexamer primer and M-MuLV Reverse Transcriptase (RNase H-). Second strand cDNA synthesis was subsequently performed using DNA Polymerase I and RNase H. Remaining overhangs were converted into blunt ends via exonuclease/polymerase activities. After adenylation of 3’ ends of DNA fragments, Adaptor with hairpin loop structure were ligated to prepare for hybridization. In order to select cDNA fragments of preferentially 370~420 bp in length, the library fragments were purified with AMPure XP system (Beckman Coulter, Beverly, USA). Then PCR was performed with Phusion High-Fidelity DNA polymerase, Universal PCR primers and Index (X) Primer. At last, PCR products were purified (AMPure XP system) and library quality was assessed on the Agilent Bioanalyzer 2100 system. Clustering and sequencing (Novogene Experimental Department). The clustering of the index-coded samples was performed on a cBot Cluster Generation System using TruSeq PE Cluster Kit v3-cBot-HS (Illumia) according to the manufacturer’ s instructions. After cluster generation, the library preparations were sequenced on an Illumina Novaseq platform and 150 bp paired-end reads were generated.

Quality control

Raw data (raw reads) of fastq format were firstly processed through in-house perl scripts. In this step, clean data (clean reads) were obtained by removing reads containing adapter, reads containing ploy-N and low quality reads from raw data. At the same time, Q20, Q30 and GC content the clean data were calculated. All the downstream analyses were based on the clean data with high quality.

Reads mapping to the reference genome

Reference genome and gene model annotation files were downloaded from genome website directly. Index of the reference genome was built using Hisat2 v2.0.5 and paired-end clean reads were aligned to the reference genome using Hisat2 v2.0.5. We selected Hisat2 as the mapping tool for that Hisat2 can generate a database of splice junctions based on the gene model annotation file and thus a better mapping result than other non-splice mapping tools.

Quantification of gene expression level

featureCounts v1.5.0-p3 was used to count the reads numbers mapped to each gene. And then FPKM of each gene was calculated based on the length of the gene and reads count mapped to this gene. FPKM, expected number of Fragments Per Kilobase of transcript sequence per Millions base pairs sequenced, considers the effect of sequencing depth and gene length for the reads count at the same time, and is currently the most commonly used method for estimating gene expression levels

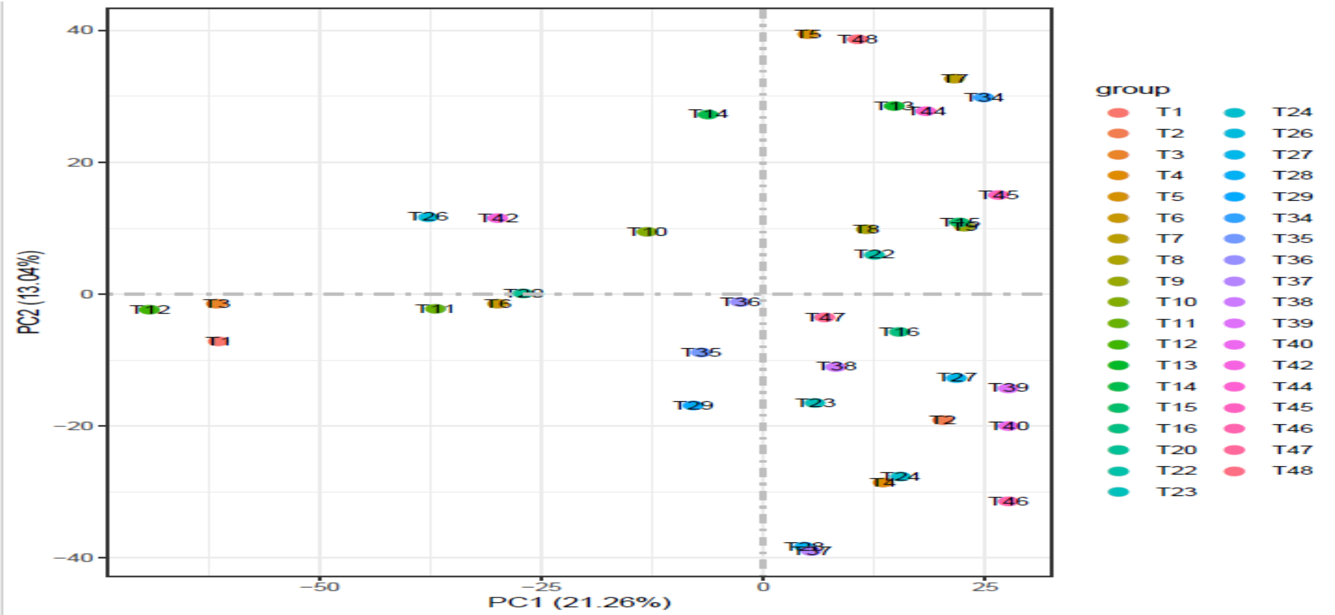

PCA outcome

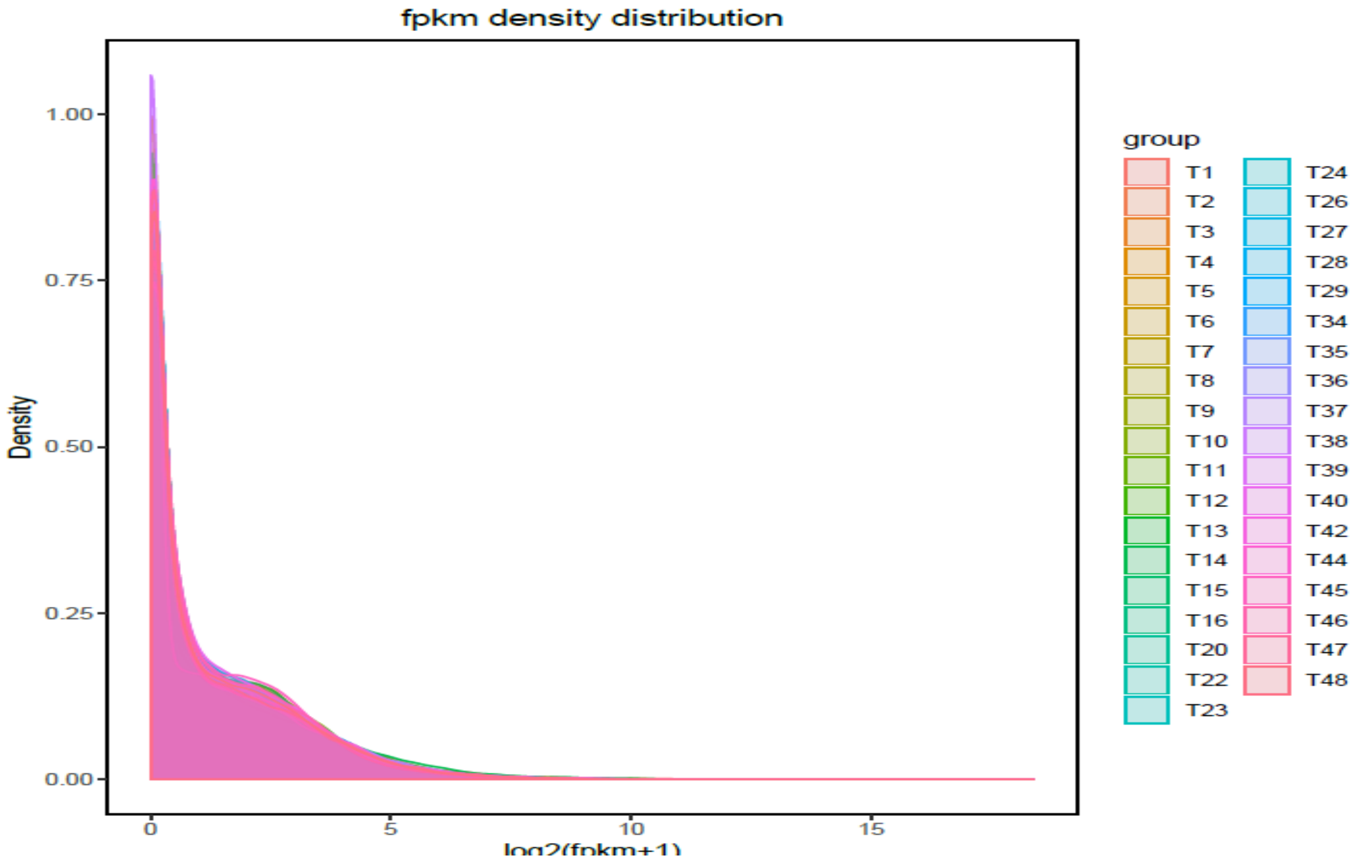

Between-sample normalization

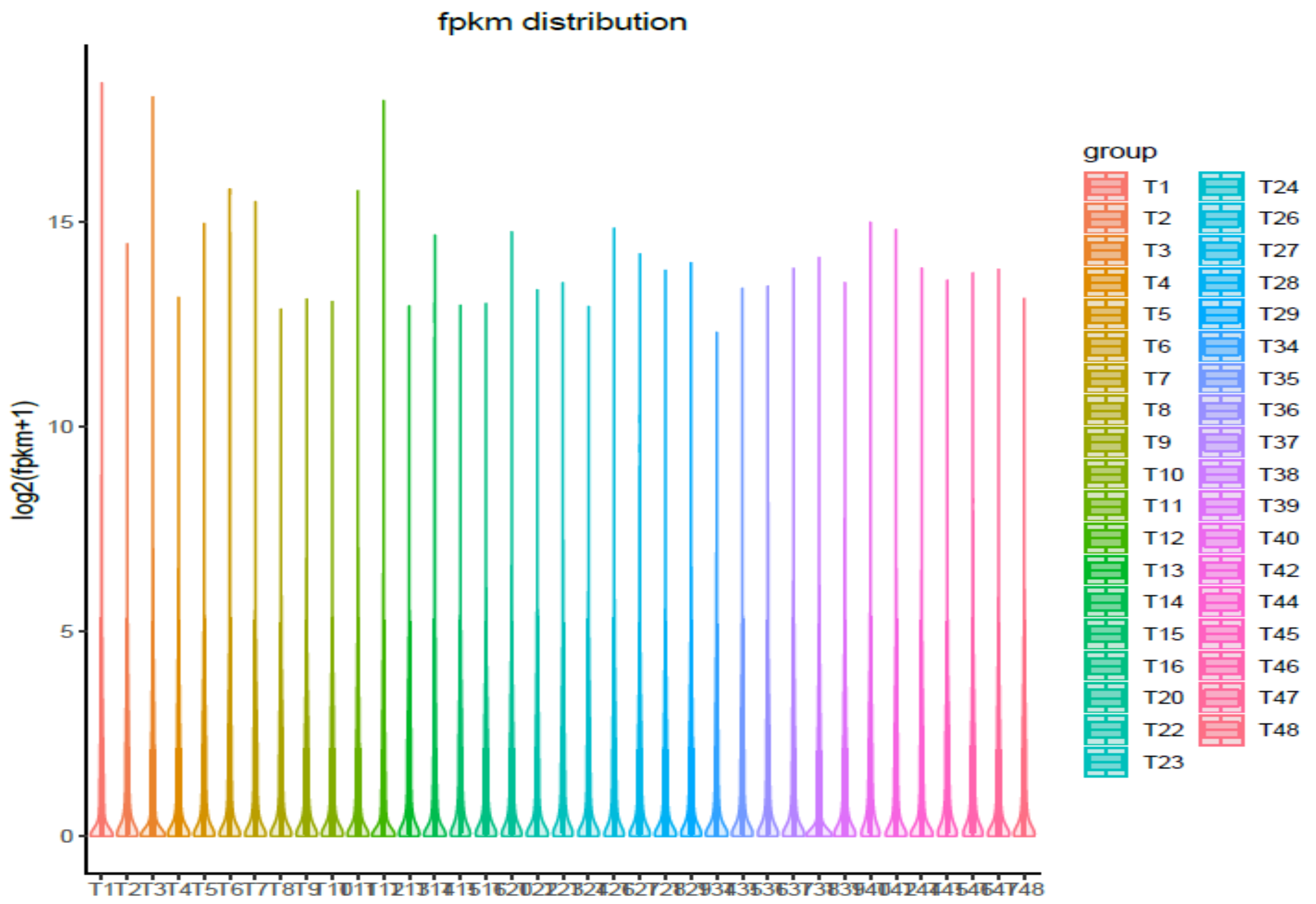
fpkm distribution
log2(fpkm+1)
0
5
10
15
group
T1
T2
T3
T4
T5
T6
T7
T8
T9
T10
T11
T12
T13
T14
T15
T16
T20
T22
T23
T24
T26
T27
T28
T29
T34
T35
T36
T37
T38
T39
T40
T42
T44
T45
T46
T47
T48

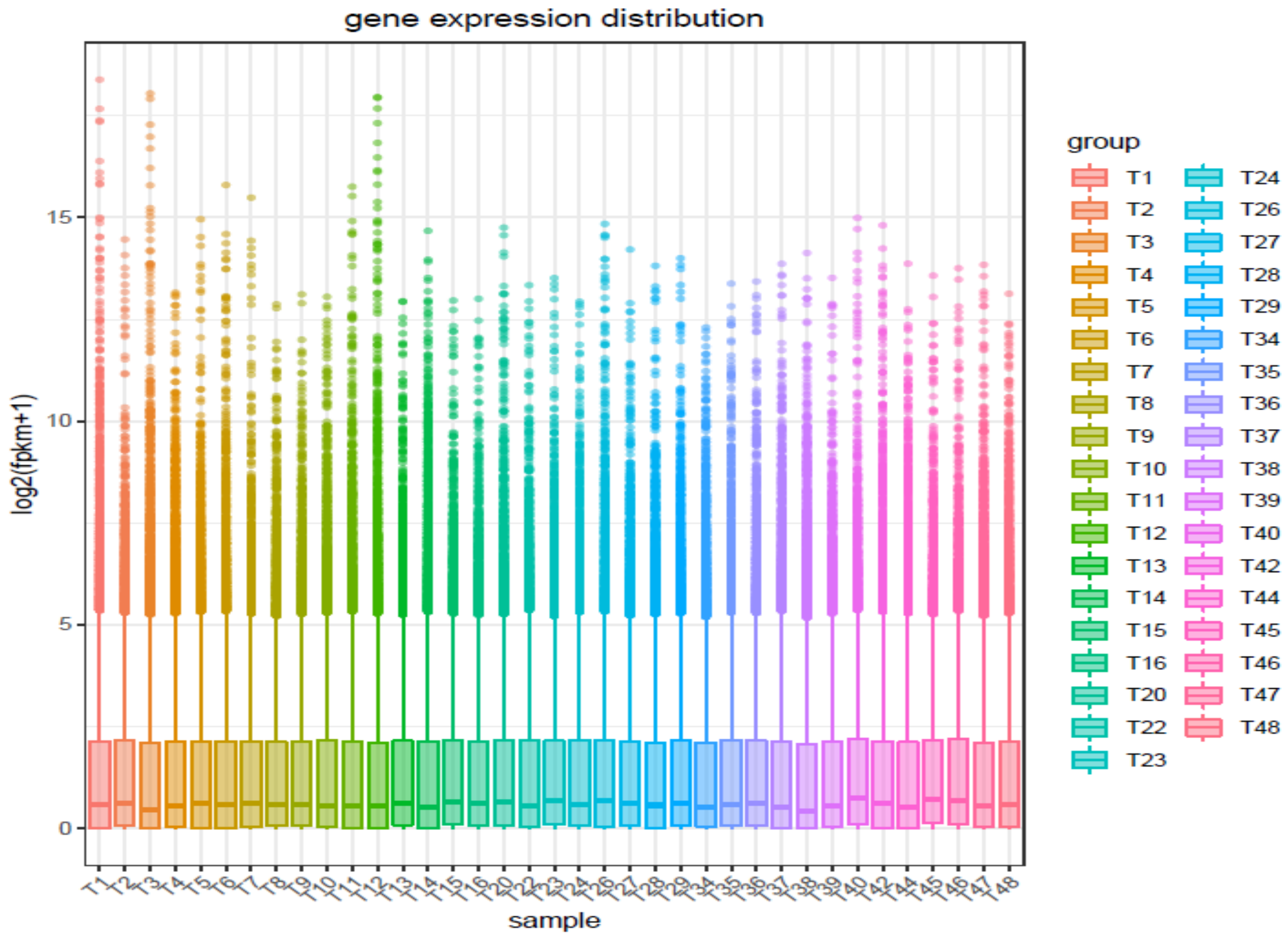
gene expression distribution
log2(fpkm+1)
0
5
10
15
sample
T1
T2
T3
T4
T5
T6
T7
T8
T9
T10
T11
T12
T13
T14
T15
T16
T20
T22
T23
T24
T26
T27
T28
T29
T34
T35
T36
T37
T38
T39
T40
T42
T44
T45
T46
T47
T48
group
T1
T2
T3
T4
T5
T6
T7
T8
T9
T10
T11
T12
T13
T14
T15
T16
T20
T22
T23
T24
T26
T27
T28
T29
T34
T35
T36
T37
T38
T39
T40
T42
T44
T45
T46
T47
T48

Sample qualities control:

sample	library	raw_reads	clean_reads	clean_bases	error_rate	Q20	Q30	GC_pct
T1	FRAS210033033-1r	46107788	42858028	6.43G	0.02	98.5	95.58	54.19
T10	FRAS210033042-1r	46205952	44640812	6.7G	0.02	98.4	95.2	51.98
T11	FRAS210033043-1r	47247440	44905390	6.74G	0.02	98.09	94.53	50.72
T12	FRAS210033044-1r	41717598	39895508	5.98G	0.02	98.27	94.9	51.3
T13	FRAS210033045-1r	44035370	41540746	6.23G	0.02	98.08	94.76	52.36
T14	FRAS210033046-1r	45370108	42366138	6.35G	0.02	98.18	94.79	51.42
T15	FRAS210033047-1r	45026142	42991732	6.45G	0.02	98.47	95.34	50.72
T16	FRAS210033048-1r	45847806	42190318	6.33G	0.02	98.35	94.85	50.75
T2	FRAS210033034-1r	45429736	43045420	6.46G	0.02	98.37	95.1	50.04
T20	FRAS210033052-1r	43579576	41366162	6.2G	0.02	98.37	95.05	47.93
T22	FRAS210033054-1r	43482886	40798144	6.12G	0.02	98.2	94.6	49.36
T23	FRAS210033055-1r	46037020	42202918	6.33G	0.02	98.21	94.5	47.87
T24	FRAS210033056-1r	49676614	45590680	6.84G	0.02	98.05	94.13	49.17
T26	FRAS210033058-2r	45620960	43835614	6.58G	0.02	98.37	95.16	51.24
T27	FRAS210033059-3r	42982954	41493098	6.22G	0.02	98.33	95.07	52.58
T28	FRAS210033060-2r	46472230	44643272	6.7G	0.02	98.2	94.79	51.7
T29	FRAS210033061-2r	45481508	43069700	6.46G	0.02	98.41	95.32	52.64
T3	FRAS210033035-1r	45116722	42042484	6.31G	0.02	98.36	95.1	49.48
T34	FRAS210033066-1r	44279456	41800940	6.27G	0.02	98.37	95.2	52.12
T35	FRAS210033067-1r	45575386	42432012	6.36G	0.02	98.14	94.71	50.76
T36	FRAS210033068-1r	43936770	40148206	6.02G	0.02	98.17	94.48	48.29
T37	FRAS210033069-1r	46123296	42466720	6.37G	0.02	98.17	94.47	48.22
T38	FRAS210033070-1r	45655248	42377208	6.36G	0.02	98.25	94.8	52.8
T39	FRAS210033071-1r	43938694	41350982	6.2G	0.02	98.21	94.61	47.47
T4	FRAS210033036-1r	47298626	44408936	6.66G	0.02	98.44	95.26	49.45
T40	FRAS210033072-1r	45414484	42296786	6.34G	0.02	98.35	94.95	46.08
T42	FRAS210033074-1r	43774564	40179786	6.03G	0.03	98.02	94.07	47.92
T44	FRAS210033076-1r	47958022	46028812	6.9G	0.02	98.31	95.07	52.22
T45	FRAS210033077-1r	45490206	43572824	6.54G	0.02	98.21	94.81	51.06
T46	FRAS210033078-1r	46023384	44464606	6.67G	0.02	98.17	94.67	49.48
T47	FRAS210033132-1r	46195720	44209612	6.63G	0.02	98.55	95.65	54.48
T48	FRAS210033133-1r	42335218	39506156	5.93G	0.02	98.24	94.87	50.76
T5	FRAS210033037-1r	45813350	42899144	6.43G	0.02	98.29	94.92	48.31
T6	FRAS210033038-1r	45660456	42514802	6.38G	0.02	98.14	94.48	48.74
T7	FRAS210033039-1r	46824376	42430844	6.36G	0.03	97.95	94.03	48
T8	FRAS210033040-1r	45656612	42068092	6.31G	0.02	98.15	94.33	48.87
T9	FRAS210033041-2r	47783124	47053736	7.06G	0.02	98.75	96.29	53.04
